# Sex differences in stress-modulated cocaine vulnerability: female rodents are more sensitive to the effects of stress exposure at different developmental stages

**DOI:** 10.3389/fnbeh.2025.1689548

**Published:** 2025-12-09

**Authors:** María Ángeles Martínez-Caballero, Claudia Calpe-López, Maria Pilar García-Pardo, M. Carmen Arenas, Carmen Manzanedo, María A. Aguilar

**Affiliations:** 1 Neurobehavioural Mechanisms and Endophenotypes of Addictive Behaviour Research Unit, Department of Psychobiology, University of Valencia, Valencia, Spain; 2 Central Institute of Mental Health, Medical Faculty Mannheim, University of Heidelberg, Mannheim, Germany; 3 Department of Psychology and Sociology, Faculty of Social Sciences, University of Zaragoza, Teruel, Spain; 4 Department of Psychobiology, University of Valencia, Valencia, Spain

**Keywords:** cocaine use disorder, sex differences, animal models, addiction, stress, females

## Abstract

**Introduction:**

Stressful life events can trigger the initiation of cocaine use, facilitate the transition to a cocaine-use disorder (CUD), and precipitate relapse. Evidence suggests that women progress more rapidly to a CUD than men. Thus, the influence of stressful life events on CUD development may differ by sex, contributing to the enhanced vulnerability seen among females. In this work, we provide a comprehensive (non-systematic) review of clinical and preclinical studies comparing the effects of cocaine and its modulation by stress in both sexes.

**Methods:**

We performed a search of the PubMed database (1986–2025) in which we combined the keywords “cocaine” and “stress” with “sex differences” or “female rat” or “female mice” or “women.” We then read the abstracts of the search results to select potentially relevant studies, which we read in full to determine if they fulfilled our criteria and to extract the relevant information.

**Results:**

Sex is often overlooked as a biological variable in preclinical and clinical research. The results of clinical studies indicate the existence of sex differences in the response to stress among individuals with CUD. Preclinical studies strongly suggest that female rodents are more vulnerable to developing addiction-like features than male rodents, particularly in the self-administration paradigm. Furthermore, exposure to stress appears to amplify the effects of cocaine, especially in females.

**Discussion:**

There is growing evidence that women and female rodents are more vulnerable to the behavioral and neurochemical changes that characterize cocaine addiction. The influence of sex should be considered in research and in the selection of strategies for preventing and treating CUD, including those targeting stress reduction.

## 1 Introduction

Cocaine is the second most commonly used illicit drug and the second most frequently cited problem drug by new treatment seekers in Europe (European Monitoring Centre for Drugs and Drug Addiction, 2024). In this context, cocaine-use disorder (CUD) currently represents a significant public health problem in developed countries. CUD arises from the interaction of biological and environmental factors, including sex/gender and stress exposure. The prevalence of cocaine use is slightly more than twice as high in men as in women (European Monitoring Centre for Drugs and Drug Addiction, 2024), but women progress more rapidly to CUD and have higher relapse rates than men (Becker et al., 2017). These trends, also observed with other illicit drugs, are known as the “telescoping effect” (Towers et al., 2023a). Research has also shown that stressful life events can trigger the initiation of cocaine use, facilitate the transition to CUD, and lead to relapse to cocaine seeking after periods of abstinence (Sinha, 2024). The available evidence regarding sex differences in cocaine vulnerability suggests that the potential effects of stress on the development of CUD also differ between males and females.

A comprehensive understanding of the mechanisms underlying the effects of stress on vulnerability to cocaine is crucial for developing effective preventive and treatment strategies for CUD. Considering the ethical and methodological limitations inherent in human studies, the use of preclinical rodent models is essential for enhancing our understanding of the role of stress in the development of cocaine addiction in both males and females. However, there is a lack of preclinical (and clinical) studies investigating sex differences in the role stress plays in modulating the effects of cocaine. Indeed, research has historically focused almost exclusively on male subjects. This oversight in considering sex as a biological variable in preclinical research can pose a significant barrier to effective translation to clinical settings (Martin et al., 2021). In this review, we have chosen to use the term “sex differences” to refer to the biological differences between males and females, and to avoid “gender,” which is a social construct that is inappropriate when discussing rodent models.

The variables sex and stress may interact synergistically to confer a particularly enhanced vulnerability to cocaine. The present work aims to provide a comprehensive (non-systematic) review of published experimental studies on sex differences in stress-modulated cocaine vulnerability and the impact of stress exposure at different developmental phases on the behavioral effects of cocaine in rats and mice, with an emphasis on female vulnerability. Firstly, we explain the methodology used to search for and select the studies included in this review. Secondly, we briefly describe the main animal models of cocaine addiction-like behavior, as well as the paradigms used to induce stress in rodents exposed to cocaine. Thirdly, we discuss the main sex differences in cocaine vulnerability and stress response in humans and rodents. Fourthly, we review the experimental evidence on how different stressful events during the prenatal period, early life, adolescence, or adulthood affect the response of female rats and mice exposed to cocaine compared to male animals. Fifthly, we examine studies focused on sex differences regarding the neurobiological mechanisms underlying the effects of stress on cocaine-related behaviors and the pharmacological modulation of these effects. Finally, we consider the implications of the results obtained in preclinical studies for the prevention and treatment of CUD in women.

## 2 Methodology of literature selection

**Figure 1:**
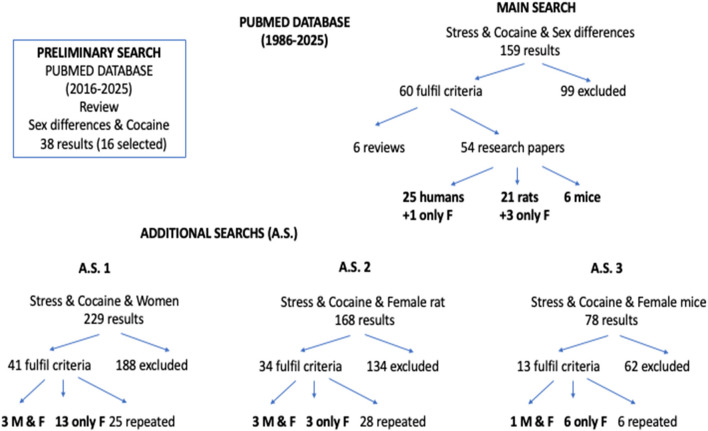
Searches in the PubMed database and the distribution of papers retrieved. The main search and additional searches (A.S.) 1–3 were performed on literature published in the last 40 years (between 1986 and 2025). A preliminary search was conducted in the PubMed database and included only review papers published from 2020 to 2025. The number of papers eventually reviewed is shown in black. M, male; F, female.

We performed a preliminary search of the PubMed database using the keywords “sex differences” and “cocaine,” filtered by paper type (review) and publication year (2020–2025). Next, we conducted a search of the PubMed database (1986–2025) combining the keywords “cocaine” and “stress” with “sex differences.” To avoid overlooking papers that included only females, we carried out three additional searches combining “cocaine” and “stress” with “female rat” or “female mice” or “women” (see Figure 1). First, we read the abstracts of the search results to select potentially relevant studies and then reviewed the entire selected works to determine if they met our criteria and to extract the relevant information. The selection criteria for studies involving animal models were that the species used were rats or mice, that the animals were treated with cocaine and exposed to a well-defined stress protocol, and that appropriate control groups were used. Exclusion criteria included the use of a different species (for example, monkeys were excluded because only two papers were found), the exclusive use of males, additional treatment with other drugs (for example, alcohol), treatment with cocaine prior to exposure to stress, and the absence of behavioral outcomes. The selection criteria for studies involving humans were as follows: the participants were men and women consuming cocaine (or crack) or with cocaine dependence or CUD; they experienced stressful life events; and appropriate control groups were used. Exclusion criteria included the use of experimental animals as subjects, the exclusive use of men, the presence of an additional drug-use disorder or problematic consumption of other illicit drugs, the presence of a psychiatric disorder, and the absence of behavioral outcomes. A total of 85 research papers and 16 reviews were ultimately selected to be covered in the present work.

## 3 Animal models of addiction-like behavior and stress

### 3.1 Animal models of cocaine addiction-like behavior

#### 3.1.1 Self-administration of cocaine

The cocaine self-administration (SA) paradigm has contributed to our understanding of the behavioral and pharmacological mechanisms of CUD. The protocol involves implanting a jugular catheter connected to a syringe that delivers cocaine when the animal makes an operant response (e.g., pressing a lever or performing a nose poke) to receive the drug under a specific schedule of reinforcement. Typically, visual or auditory conditioned stimuli are associated with cocaine administration to model the presence of drug-associated cues in human use. Different reinforcement schedules can be applied. In fixed ratio (FR) and fixed interval (FI) schedules, behavior is reinforced at a constant rate or at each predetermined unit of time. For example, in FR2, two operant responses are required from a rodent to obtain the reinforcer (cocaine), whereas in FI2, the operant response may be reinforced every 2 min. In a progressive ratio (PR) schedule, the number of responses required from a rodent to obtain the reinforcer increases progressively after each instance of reinforcement until a breaking point is reached (the highest number of responses made by an animal to obtain the drug reinforcer). This breakpoint reflects the animal's motivation to obtain the drug.

#### 3.1.2 Conditioned place preference induced by cocaine

The rewarding properties of cocaine, an important aspect of CUD, can be assessed using the place conditioning paradigm, a Pavlovian learning task in which cocaine (the unconditioned stimulus) is explicitly paired with salient contextual conditioned stimuli that subsequently acquire positive incentive salience. Consequently, the process of place conditioning with cocaine induces a conditioned place preference (CPP) for the context previously associated with this drug. The approach behavior of rodents to the cocaine-paired context has been shown to model the tendency of individuals with CUD to approach contextual stimuli associated with previous instances of drug use, often resulting in the reinstatement of previously extinguished cocaine-seeking behavior.

#### 3.1.3 Extinction and reinstatement protocols

The SA and CPP paradigms have been shown to be effective models for studying relapse (Aguilar et al., 2009; Shaham et al., 2003). Following the acquisition phase, subjects undergo extinction of the SA or CPP (without drug administration) until the cocaine-seeking behavior or place preference decreases or disappears. When the extinction criterion is reached, the ability of drug priming, drug-associated cues, or stress exposure to induce reinstatement of the SA or CPP is assessed. The reinstatement model of cocaine relapse has demonstrated high face validity (i.e., the resumption of regular patterns of cocaine use after a period of abstinence is modeled by the resumption of cocaine SA or CPP after a period of abstinence) and criterion validity (i.e., a similar stimulus triggers the reappearance of cocaine-seeking behavior during abstinence in humans and animals, and effective interventions to prevent reinstatement in rodents also reduce relapse in humans) (Bossert et al., 2013; Epstein et al., 2006). Voluntary abstinence can also be induced in animals through contingent punishment of the operant response or by administering a footshock in the drug-associated compartment to model the negative consequences of drug use (Deroche-Gamonet et al., 2004). Alternatively, animals can undergo a period of enforced abstinence to model the progressive increase in cue-induced craving (incubation effect) that occurs following the initiation of abstinence in humans with CUD (Tran-Nguyen et al., 1998).

#### 3.1.4 Locomotor activation and behavioral sensitization

According to the psychostimulant theory, the psychomotor activating and reinforcing effects of cocaine are mediated by the same mechanisms (activation of striatal dopaminergic receptors) and reflect its potential for dysregulated use (Wise and Bozarth, 1987). Locomotor activation includes approach behavior, forward locomotion, traveled distance, ambulation, rearing, rotational behavior, and stereotypies. Repeated administration of cocaine can lead to sensitization, also known as behavioral sensitization, characterized by a progressive increase in the effects of the drug. Behavioral sensitization is a key indicator of the sensitization of drug “wanting” (Robinson and Berridge, 2025), a significant aspect of CUD that reflects the initial transition from occasional to dysregulated use (Steketee and Kalivas, 2011).

### 3.2 Animal models of stress used in rodents exposed to cocaine

#### 3.2.1 Prenatal stress induced by restraint of gestational dams

In this model, pregnant female rats are subjected to restraint during the final week of gestation. The restraint protocol typically commences between embryonic days 11 and 15 and continues until birth. This protocol involves placing pregnant rats in a Plexiglass restrainer for 45–60 min, three times per day (Bagley et al., 2019; Kippin et al., 2008; Thomas and Becker, 2019; Thomas et al., 2009). Prenatal stress has been shown to result in an anhedonic state in offspring, which can contribute to maladaptive motivated behavior.

#### 3.2.2 Early-life stress induced by neonatal isolation or maternal separation

In the protocol for neonatal isolation-induced stress, each pup is removed from the dam for 1 h per day, usually from postnatal day (PND) 2 to 9. The pup is then placed individually in another container, such as a glass jar with bedding, which is in turn placed in a plastic container filled with heated water (32–34 °C) to maintain an appropriate body temperature (Ganguly et al., 2019; Lynch et al., 2005). Alternatively, the pup is placed in a plastic container without bedding in a heated (30 °C) chamber with white noise to mask the calls of other pups (Kosten et al., 2004, 2006). The consequences of neonatal isolation have been observed in puppies at PND10 (Kosten et al., 2003), during adolescence (Ganguly et al., 2019), and in adulthood (Kosten et al., 2006; Lynch et al., 2005).

A variety of protocols involving maternal separation have been employed to assess the consequences of stress. In one such protocol, whole litters of pups are separated from the nest (and parents) on 10 occasions between PND 5 and 20 (Matthews et al., 1999) or for 1 h daily from PND 1 to 13 (Kikusui et al., 2005). To prevent hypothermia, the litter is placed in a wire basket inside an incubator or in a plastic container, with a layer of nest shavings placed over a heating pad set at 35 °C. In alternative protocols, the pups remain in their home cages while the mother is moved to another cage for 24 h from PND 9 to PND 10 (Bis-Humbert and García-Fuster, 2021; Bis-Humbert et al., 2021a,b). In mice, the combination of repeated maternal separation (4 h per day on postnatal days 2–5 and 8 h per day on postnatal days 6–16) with early weaning (PND 17) is used as an animal model of childhood adversity (Vetulani, 2013). During the process of maternal separation, the pups are kept in their home cages with a heating blanket (set to between 32 and 34 °C) to ensure their thermoregulation.

Another protocol involving an unstable maternal environment is repeated cross-fostering (Di Segni et al., 2017, 2019, 2020), in which pups are fostered by four adoptive mothers from PND 1 to PND 4 and remain with the last adoptive mother until weaning. Pups from the same litter spend the first postnatal day (PND 0) with their biological mother. On PND 1, the entire litter is introduced into the home cage of a different dam whose pups have been moved to another adoptive mother. This procedure is repeated daily until the pups are placed with the fourth adoptive mother (PND 4). Repeated cross-fostering has been shown to modify sensitivity to cocaine in adult female mice (Di Segni et al., 2017).

#### 3.2.3 Exposure to social defeat stress during adolescence or adulthood

Social confrontation is defined as a form of social interaction between two conspecific individuals who confront each other, typically using the resident/intruder paradigm. This confrontation involves a dominant individual (the resident in the home cage, which exhibits threatening and attack behaviors) and a submissive individual (the intruder or loser, which displays avoidance, fleeing, and defensive/submissive behaviors). Several social defeat protocols have been extensively utilized in male rodents to model social stress and study its underlying neural mechanisms (Miczek et al., 2008; Vannan et al., 2018). In female rodents, social defeat can be induced by territorial or maternal aggression (Shimamoto, 2018). Research indicates that stress induced by social defeat can amplify the rewarding effects of cocaine and facilitate addictive behaviors. The extent of these effects varies depending on the intensity, duration, and frequency (acute, intermittent, or chronic) of the defeat episodes.

In one protocol of social defeat, male and female rats are exposed to aggressive attacks by a same-sex opponent (a larger aggressive male or a lactating female, respectively), culminating in the defeat of the experimental animal. Acute exposure to defeat is frequently used to evaluate the effects of social stress on the reinstatement of cocaine-seeking behavior in mice (Ribeiro Do Couto et al., 2009). In other studies, the episodes of defeat are experienced intermittently on four separate occasions over the course of 1 week (Haney et al., 1995) or every 72 h in rats (Holly et al., 2012) and mice (Calpe-López et al., 2020; García-Pardo et al., 2019). The most frequently employed protocol of social defeat is chronic social defeat stress, in which rodents are exposed to defeat episodes on a daily basis (typically for 10 or 21 consecutive days). A modified resident-intruder paradigm has been employed for female rats, in which lactating dams with pups (PND 3–12) serve as the aggressive stimulus. In the 21-day experiment, female rats are exposed to two daily episodes. The first episode consists of a 30-min direct confrontation between the rats, followed by a threat period in which they are placed in a wire mesh protective cage until the next direct confrontation (Shimamoto et al., 2015, 2018).

The most frequently used protocol of social defeat in female rodents is vicarious social defeat stress, in which female mice experience one episode of vicarious defeat on 10 consecutive days (Iñiguez et al., 2018) or over 4 days separated by intervals of 72 h (Martínez-Caballero et al., 2024). In each episode, the experimental female mouse witnesses a male mouse of the same strain being defeated in an agonistic encounter with a more aggressive male mouse. At the start of each episode, a female mouse is placed in the home cage of an aggressive male mouse (resident), with the two mice separated by a perforated methacrylate wall. Subsequently, an intruder male mouse is introduced into the compartment of the resident male, and the two males are allowed to confront each other for a period of 5 min. The perforated methacrylate wall ensures the perception of olfactory and chemosensory stimuli from the defeat episode, which induces emotional and psychological stress in the female mice (Iñiguez et al., 2018).

#### 3.2.4 Other protocols of stress in adolescent or adult rodents

It is important to note that acute or chronic exposure to various environmental conditions or events can induce stress in rodents. In the social isolation-induced stress protocol, rats or mice are isolated during adolescence (e.g., from PND 22 to 42) and then grouped (Walker et al., 2022a, b), isolated throughout the entire adolescent period (from PND 21) until adulthood (Deutschmann et al., 2022; Fosnocht et al., 2019), or isolated during late adolescence (Westenbroek et al., 2013) or in adulthood (Benite-Ribeiro et al., 2014).

Other methods of inducing stress in adolescent or adult rodents include restraint (immobilization) or forced swimming in an inescapable environment. Acute or repeated exposure to immobilization using plastic semi-cylindrical restrainers is frequently used to study the effects of stress on the reinstatement of cocaine-seeking behavior. The swim stress protocol involves placing rodents in a cylindrical pool filled with 30 °C water for 5–10 min (Bourke and Neigh, 2011).

Unpredictable, intermittent electric footshock stress has also been used to induce stress before the acquisition of self-administration (SA) (Morales-Silva et al., 2024), to punish previously acquired cocaine SA (Gaulden et al., 2025), or to induce reinstatement of cocaine seeking (McFarland et al., 2004). In addition, pharmacological stress can be induced by administering drugs related to the hypothalamic–pituitary–adrenal (HPA) axis, such as corticotropin-releasing factor (CRF) or the alpha-2 adrenergic receptor antagonist yohimbine.

The combination of different stressful events has also been applied in adolescent and adult rodents. For instance, female rats exposed to social isolation (PND 25–29) along with swim stress (PND 25 and 29), restraint stress (PND 26 and 28), footshock stress (PND 27), and 24 h of light (PND 26 and 28) have exhibited heightened cocaine-induced impulsivity (Paine et al., 2021). In adolescent female Wistar rats, the combination of social isolation (from PND 35–49) with 6 days of social defeat (by a female Long Evans rat) and 6 days of restraint (60 min) on an alternating schedule lasting 12 days resulted in decreased sucrose consumption and reduced corticosterone release induced by stress (Bourke and Neigh, 2011; Rowson et al., 2018). Finally, adolescent rats (PND 21–35) exposed to a combination of stressful events (social isolation, food restriction, forced swim, restraint, and predator odor) were reported to display addiction risk traits, an effect that was more pronounced in female rats (Hynes et al., 2018).

## 4 Sex differences in cocaine vulnerability and response to stress

### 4.1 Sex differences in cocaine vulnerability

A series of excellent reviews have been published over the last 10 years regarding sex differences in vulnerability to various addictive drugs, including cocaine (Becker, 2016; Becker et al., 2017; Dos Anjos Rosário et al., 2022; Hersey et al., 2023; Knouse and Briand, 2021; Kokane and Perrotti, 2020; Nicolas et al., 2022; Peart et al., 2022; Torres, 2022). Although this issue is beyond the scope of the present work—which focuses on sex differences in stress-modulated cocaine vulnerability—it is relevant to begin by describing the main findings concerning sex differences in CUD and the behavioral effects of cocaine in rodents. In addition, the biological substrates of these sex differences are reviewed in this section.

#### 4.1.1 Clinical studies

No clear sex differences have been reported regarding the age of first cocaine use, frequency of exposure to the drug, or transition to CUD. However, some studies involving individuals with CUD seeking treatment have suggested that women are more likely to report pleasure and positive mood in response to cocaine (Evans, 2007), progress more quickly to cocaine addiction, and maintain shorter abstinence periods (Haas and Peters, 2000; McCance-Katz et al., 1999; White et al., 1996). They also report higher levels of desire to use cocaine than men (for a review, see Hersey et al., 2023). Furthermore, women with CUD face specific challenges during abstinence, experiencing higher levels of negative affect (Griffin et al., 1989) and cravings induced by drug-associated cues and stress (see reviews by Martin et al., 2021, and Peart et al., 2022), along with more pronounced withdrawal symptoms (Hudson and Stamp, 2011) than men, which may contribute to an increased tendency to relapse. In addition, sex differences in early-abstinent crack cocaine users have been reported. Research by Rebelatto et al. (2022) demonstrated that women exhibit a more rapid progression to crack-use disorder. Furthermore, they experience higher rates of trauma and stress-related disorders, a greater number of problems (including childcare issues, criminal involvement, and work-related difficulties), and a reduced level of social support (Sanvicente-Vieira et al., 2019). However, a recent review of clinical studies did not support the hypothesis that women are more vulnerable to psychostimulant craving and relapse (for review, see Nicolas et al., 2022). In total, 17 studies were conducted to examine sex differences in cocaine craving; five found greater craving in women, one observed higher craving in men, and 11 did not report any sex differences. Similarly, of the 10 studies that examined relapse to cocaine seeking, only one reported higher relapse in women (although they were cocaine and heroin users), while five reported the opposite, and four did not find sex differences at 6-month follow-up or later (see Table 1; two studies involving individuals with CUD and opioid-use disorder were not included in the table).

**Table 1:** Clinical studies on sex differences in craving and relapse in individuals with CUD.

**Craving/Relapse**	**Evaluation**	**Male**	**Female**	**References**	**Additional information**
Spontaneous drug craving	Craving questionnaire of six items			Elman et al., 2001	12 h of abstinence
	Craving/Distress/Mood scale			Waldrop et al., 2010	2 days of abstinence
	Brief version of Cocaine Craving Questionnaire			Volkow et al., 2011	Non abstaining users
	A 10-point visual analog scale			Fox et al., 2006	1 week of abstinence
	Brief version of Cocaine Craving Questionnaire			Fox et al., 2013	1 week of abstinence
	Original and brief Cocaine Craving Questionnaire			Paliwal et al., 2008	90 days of abstinence
Drug-induced craving	Multidimensional craving questionnaire and Addiction Severity Index			Elman et al., 2002	10 hours of abstinence
	A 10-point visual analog scale			Evans et al., 1999	Non abstaining users
Cue-induced craving	Craving/Distress/Mood scale			Waldrop et al., 2010	2 days of abstinence
	Brief version of Cocaine Craving Questionnaire			Volkow et al., 2011	Non abstaining users
	A 10-point visual analog scale			Fox et al., 2006	1 week of abstinence
	A 10-point visual analog scale			Potenza et al., 2012	At least 2 weeks of abstinence
	A 10-point visual analog scale			Fox et al., 2014	14–21 days of abstinence
	A 10-point visual analog scale			Robbins et al., 1999	Long-term outpatient treatment
Stress-induced craving	Craving/Distress/Mood scale			Waldrop et al., 2010	Social stressor; 2 days of abstinence
	A 10-point visual analog scale			Moran-Santa Maria et al., 2014	Yohimbine; 2–3 days of abstinence
	A 10-point visual analog scale			Back et al., 2005	Psychological or physical stressors. At least 3 days of abstinence
	Craving/Distress/Mood scale			Brady et al., 2009	CRF; 90 days of abstinence
	A 10-point visual analog scale			Li et al., 2005	Script-guided stressful situation. At least 2 weeks of abstinence
Relapse	Supervised urine toxicology			Kosten et al., 1993	21 days of treatment
	Modified version of Addiction Severity Index			Kosten et al., 1993	6-month follow-up
	Supervised urine toxicology			Bashiri et al., 2017	3 months of treatment
	Self-reports and supervised urine toxicology			Gallop et al., 2007	6 months of treatment
	Addiction Severity Index and self-reports			Weiss et al., 1997	6-month follow-up
	Supervised urine toxicology			Sterling et al., 2004	9-month follow-up
	Psychological arousal and < 3 months of sustained abstinence			Negrete and Emil, 1992	12-month follow-up
	Supervised urine toxicology and blood samples			McKay et al., 2003	12-month follow-up

Pink, higher in females; blue, higher in males; grey, no sex differences.

##### 4.1.1.1 Neurobiological substrates of cocaine reward between sexes in clinical studies

Imaging studies have demonstrated sex differences in brain responses to cocaine and cues associated with cocaine in individuals with cocaine dependence (see Nicolas et al., 2022, for a comprehensive overview). In these studies, videos, pictures, or scripts were presented to subjects to induce craving and measure changes in brain activity (e.g., glucose utilization) triggered by neutral or cocaine cues. Levels of subjective craving were also evaluated. The activation of brain areas in response to cocaine-associated cues or the degree of activation varies between men and women (see Table 2). However, no differences in cue-induced cocaine craving were observed between the sexes (Joseph et al., 2019; Kilts et al., 2004; Kober et al., 2016; Zhang et al., 2020). One study revealed a decrease in whole-brain activation among female subjects, while male subjects demonstrated an increase. Furthermore, compared to men, women exhibited increased cue-related deactivation in brain regions associated with executive control (Volkow et al., 2011), and the activation of the dorsal anterior cingulate cortex and nucleus accumbens (NAcc) in response to emotional cues was found to be negatively correlated with the duration of regular cocaine use in female subjects with CUD. However, this correlation was not observed in male subjects with CUD. These findings suggest that, over time, female subjects become less sensitive to aversive stimuli, including the adverse consequences associated with cocaine use. This phenomenon may explain the telescoping effect observed in female subjects (Tap et al., 2024). Despite the methodological limitations of these studies (e.g., low sample sizes and active cocaine use vs. abstinence), their results suggest that the brain circuits involved in cocaine craving differ between men and women.

**Table 2:** Imaging studies on the activation of brain areas in response to cocaine-associated cues in men and women with CUD.

**Comparison between sexes**	**Male**	**Female**	**References**	**Additional information**
Whole brain activation			Volkow et al., 2011	Activation induced by cues
Activation of brain regions associated with executive control (prefrontal, cingulate, inferior parietal, thalamus)			Volkow et al., 2011	Activation induced by cues
Activation of the middle frontal gyrus			Cousijn et al., 2021	Activation during a working memory task
**Comparison with controls of the same sex**				
Activation of the dorsal anterior cingulate cortex and nucleus accumbens	No correlation observed	Negative correlation with the duration of cocaine use	Tap et al., 2024	Activation induced by emotional cues
Gray matter volume left anterior insula and lingual gyrus	No differences respect to male control	Larger compared to female control	Rabin et al., 2022	
Gray matter volume in right hippocampus	Negative correlation with cocaine use duration	No correlation observed	Rabin et al., 2022	
Activation of the inferior frontal gyrus, insla and putamen	No correlation observed	Negative correlation with the cocaine use severity in female	Cousijn et al., 2021	Activation during a working memory task

Pink, higher in females; blue, higher in males; grey: no sex differences.

Sex-specific differences have also been reported regarding gray matter volume in limbic areas and prefrontal cortex (PFC) functioning in individuals with CUD (see Table 2). The left anterior insula and left lingual gyrus were found to be larger in women with CUD compared to controls, while the volume of the right hippocampus was negatively associated with cocaine duration in men with CUD. These findings suggest that the mechanisms underlying cocaine addiction differ between women and men (Rabin et al., 2022). In addition, cocaine-dependent women demonstrated impaired executive functioning compared to cocaine-dependent men, as evidenced by an increased number of omission errors and prolonged reaction times (Moran-Santa Maria et al., 2016). Similarly, female cocaine users exhibited heightened activation in the middle frontal gyrus during a working memory task compared to male users. Additionally, activation of the inferior frontal gyrus, insula, and putamen was negatively correlated with the severity of cocaine use in female users. These findings suggest that impairments of the PFC play a more significant role in the transition from recreational use to CUD in women than in men (Cousijn et al., 2021).

##### 4.1.1.2 Hormonal influences on cocaine vulnerability

A significant factor contributing to these sex differences is the level of gonadal hormones (Evans and Foltin, 2010). Research indicates that the hedonic effects of cocaine (Martin et al., 2021) and crack (Evans et al., 2002; Sofuoglu et al., 1999), as well as the craving induced by exposure to drug-associated cues and stress, are higher among women during the follicular phase of the menstrual cycle (characterized by high estradiol levels) than during the luteal phase (characterized by high progesterone levels) (for review see Martin et al., 2021; Peart et al., 2022; Torres, 2022). In addition, progesterone (which reduces the effects of estradiol) has been shown to attenuate the subjective effects of smoked cocaine in women but not in men (Evans and Foltin, 2006).

#### 4.1.2 Preclinical studies

As shown in Table 3, a significant body of research using preclinical models indicates that female rodents demonstrate a heightened susceptibility to the reinforcing effects of cocaine compared to males (for a review, see Torres, 2022).

**Table 3:** Sex differences in the effects of cocaine in preclinical models of addictive behavior.

**Paradigm**	**Animal model of**	**Male**	**Female**	**References**	**Additional information**
Acquisition of SA	Primary reinforcing effects			Roth and Carroll, 2004	Faster acquisition
				Lynch and Carroll, 1999	Faster acquisition
				Martini et al., 2014	Faster acquisition
				Castro-Zavala et al., 2020	Faster acquisition
				Castro-Zavala et al., 2021	Faster acquisition
				Lynch, 2008	Adolescent rats
				Griffin et al., 2007	More challenging PR, 0.3 mg/kg
				Griffin et al., 2007	1 mg/kg
				Haney et al., 1995	No clear sex-differences
				Thomas et al., 2009	No clear sex-differences
				Gaulden et al., 2025	No clear sex-differences
Cocaine intake in SA	Excessive drug use			Lynch and Taylor, 2004	Higher in long-access sessions
				Lynch et al., 2005	Higher in long-access sessions
				Moschak et al., 2023	Higher in long-access sessions
				Templeton et al., 2023	Higher in long-access sessions
				Griffin et al., 2007	More challenging PR, 0.3 MG/KG
Escalation of SA	Loss of control			Roth and Carroll, 2004	Higher escalation
Response in progressive ratio	High motivation for cocaine			Lynch and Taylor, 2004	Higher response
				Martini et al., 2014	Higher response
				Kawa and Robinson, 2019	Higher response (intermittent/extended protocols)
				Towers et al., 2021	Higher response (intermittent/extended protocols)
				Quigley et al., 2021	Higher breaking point
Extinction of SA	Continued use, compulsivity			Anker and Carroll, 2010	Higher resistance
				Lynch et al., 2005	Higher response in the first extinction session
Punishment of SA	Continued use even when causes problems			Towers et al., 2021	Greater resistance to punishment of SA
Priming-induced reinstatement	Relapse induced by drug reexposure			Doncheck et al., 2020	Higher
				Lynch et al., 2005	No clear sex-differences
				Kawa and Robinson, 2019	No clear sex-differences
Cue-induced seeking	Relapse induced by drug-associated cues			Morales-Silva et al., 2024	Higher in females stressed before SA acquisition
Cue-induced reinstatement				Lynch et al., 2005	No clear sex-differences
				Kawa and Robinson, 2019	No clear sex-differences
Stress-induced reinstatement	Relapse induced by stress			Anker and Carroll, 2010	Yohimbine before reinstatement test
Vulnerability to incubation	Cocaine craving after abtinence			Towers et al., 2023b	Faster incubation in early withdrawal
				Corbett et al., 2021	Higher vulnerability
				Johnson et al., 2019	Higher vulnerability
				Kerstetter et al., 2008	Higher vulnerability
				Madangopal et al., 2019	Higher vulnerability
				Nicolas et al., 2019	Higher vulnerability
Presence of addiction like-criteria				Thomas and Becker, 2019	Fulfill more criteria (particularly after stress)
				Towers et al., 2021	Fulfill more criteria
Acquisition of CPP	Conditioned rewarding effects			Unterwald et al., 2013	No clear sex-differences
				Unterwald et al., 2013	Higher at lower doses
Extinction of CPP				Hilderbrand and Lasek, 2014	More resistant
Locomotor sensitization	Sensitization to cocaine reward			Sershen et al., 1998	No clear sex-differences, depend on the strain
				Sershen et al., 1998	Higher in C57BL/6B females
				Milivojevic et al., 2016	Higher in C57BL/6B females
				Chapp et al., 2023	Higher in C57BL/6B males
Impulsivity	Impulsivity			Paine et al., 2021	High in females exposed to stress and cocaine

SA, Self-administration of cocaine; CPP, conditioned place preference induced by cocaine. Pink indicates that the effect was greater in females than in males; blue indicates that the effect was stronger in males than in females; gray indicates no clear sex differences.

Following short-access (1–2 h) to cocaine SA, female mice acquired SA at a faster rate in some studies (Castro-Zavala et al., 2020; Martini et al., 2014), but no sex differences were observed in other studies with mice (Griffin et al., 2007) or rats (Gaulden et al., 2025; Haney et al., 1995; Roth and Carroll, 2004; Thomas et al., 2009). On the other hand, after long-access sessions (5–24 h), females showed higher cocaine intake (Lynch and Taylor, 2004; Lynch et al., 2005; Moschak et al., 2023; Templeton et al., 2023), escalated cocaine use more rapidly (Lynch and Carroll, 1999; Roth and Carroll, 2004), and demonstrated a more rapid and greater increase in motivation for cocaine (Towers et al., 2021). These sex differences were also observed in adolescent female rats (Lynch, 2008) and with intermittent long-access protocols (Kawa and Robinson, 2019).

Adolescent female rats also acquired cocaine SA under PR schedules faster than their male counterparts (Lynch, 2008), and both adult female mice and rats responded more than males in a PR schedule (Lynch and Taylor, 2004; Martini et al., 2014) and displayed a higher breaking point for cocaine (Quigley et al., 2021). Conversely, male mice responded more and consumed more cocaine than females in a PR schedule with a low dose of cocaine (0.3 mg/kg/infusion) (Griffin et al., 2007).

Regarding extinction and reinstatement of SA, female rats tended to respond at higher levels during the initial extinction sessions than males (Lynch et al., 2005) and showed greater resistance to extinction (Anker and Carroll, 2010). Females were more vulnerable to priming-induced reinstatement after short-access cocaine SA training (Doncheck et al., 2020), although no sex differences in the reinstatement of SA induced by priming or cocaine-paired cues were reported in studies using intermittent or long-access protocols of cocaine SA (Kawa and Robinson, 2019; Lynch et al., 2005). Additionally, females may be less vulnerable to cue-induced reinstatement than males when the conditioned stimulus has low motivational salience (Fuchs et al., 2005). In comparison to males, female rats have also shown an accelerated time course of incubation, with elevated cocaine craving during early withdrawal (Towers et al., 2023b), and were more vulnerable to incubation after a period of forced abstinence ranging from 14 to 200 days (Corbett et al., 2021; Johnson et al., 2019; Kerstetter et al., 2008; Madangopal et al., 2019; Nicolas et al., 2019). Female rats trained with short-access protocols of SA were more sensitive to reinstatement of SA induced by several stressful stimuli such as yohimbine, CRF, and footshocks (Anker and Carroll, 2010; Buffalari et al., 2012; Connelly et al., 2020).

Other studies have failed to detect clear sex differences in cocaine-induced CPP (Unterwald et al., 2013), although male mice were more sensitive than female mice to acquiring CPP at lower doses (Calpe-López et al., 2020; Martínez-Caballero et al., 2024; Unterwald et al., 2013) and more resistant to extinction (Hilderbrand and Lasek, 2014). Similarly, no sex differences were observed in mice regarding locomotor sensitization (Chapp et al., 2023; Sershen et al., 1998). Some studies have reported greater locomotor and sensitization responses in female C57BL/6B mice (Milivojevic et al., 2016; Sershen et al., 1998), while others have observed the opposite effect, with male C57BL/6B mice showing greater sensitization (Chapp et al., 2023). These divergent results may be due to methodological differences between studies, such as variations in cocaine dosage and administration schedules.

In summary, no clear sex differences were reported in the acquisition or priming- and cue-induced reinstatement of cocaine SA with short-access protocols. However, females were found to be more sensitive to the reinforcing effects of cocaine and to priming- and cue-induced reinstatement after intermittent or long-access SA sessions. In addition, exposure to stress exacerbated these sex differences. More addiction-like criteria were observed among females compared to males exposed to stress prior to cocaine in an extended regimen of cocaine self-administration (Thomas and Becker, 2019). Furthermore, females were more vulnerable to stress-induced reinstatement. Finally, female rats exhibited greater resistance than males to the punishment associated with long-access cocaine SA (Towers et al., 2021), suggesting more compulsive use than males, while the opposite was observed after shorter-access sessions of cocaine SA (Edson and Ball, 2022).

##### 4.1.2.1 Neurobiological substrates of cocaine reward between sexes in preclinical studies

The existence of sex differences in CUD may be associated with the differences in the neurobiological substrates of cocaine reward between sexes observed in preclinical studies (see Table 4). Cocaine blocks the dopamine (DA), serotonin (SER, also known as 5-hydroxytryptamine, 5-HT), and noradrenaline (NA) transporters (DAT, SERT, and NAT, respectively), inhibiting the reuptake of these extracellular monoamines. This results in an increase in synaptic DA, 5-HT, and NA in several brain areas. The main structures involved in the development of cocaine addiction are dopaminergic areas such as the medial PFC (mPFC), the mesolimbic system (including the ventral tegmental area (VTA) and NAcc), and the nigrostriatal system (including the substantia nigra and the dorsal striatum). The mPFC is associated with executive functioning and is implicated in the development of psychiatric disorders. The mesolimbic system is involved in motivated behaviors and the rewarding effects of cocaine. The dorsal striatum, which is associated with habit formation, is responsible for the shift from voluntary to compulsive drug use (for a more detailed description, see Torres, 2022). Research has identified sex differences in the mesocorticolimbic DA system, with gonadal hormones playing a critical role in regulating DA transmission (Eck and Bangasser, 2020). It has been proposed that estradiol and progesterone exert opposite effects on DA neurotransmission in the mesolimbic system (Nicolas et al., 2022). As previously mentioned, these hormones, respectively, increase or decrease cocaine craving and relapse. Female mice have higher levels of DAT in the NAcc (Kikusui et al., 2005), and cocaine-induced increases in DA levels and downregulation of the DAT were found to be most significant in female rats during the estrous phase (for a review, see Hersey et al., 2023; Kokane and Perrotti, 2020). Ovariectomy in female rats led to a reduction in VTA firing rates and cocaine-induced DA release in the NAcc, while estradiol replacement restored these effects (Cummings et al., 2014; Zhang et al., 2008). Neuron firing rates in the VTA and cocaine-induced DA release were higher during estrus than in diestrus (Calipari et al., 2017). In female mice, the administration of estradiol or an agonist of the estradiol receptor β induced cFOS expression in the NAcc (Satta et al., 2018) and increased cocaine-induced DA release in this structure (Yoest et al., 2019). Microinjections of estradiol in the medial preoptic area of adult rats also enhanced cocaine-induced DA release in the NAcc and cocaine-induced CPP, thereby demonstrating that hormonal signaling can exert an indirect influence on the brain reward system (Robison et al., 2018a; Tobiansky et al., 2016). Conversely, acute (but not chronic) cocaine exposure has been shown to regulate a greater number of genes in the brain reward system in male rodents than in females (Walker et al., 2022b). In addition to DA, sex differences in the response of serotonin neurons were observed after cocaine administration, and ovarian hormones contributed to this effect (Dos Anjos Rosário et al., 2022). In female rats, the administration of cocaine increases serotonin release in the NAcc (Shimamoto et al., 2011), and drug-seeking behavior during the initial abstinence period following cocaine SA involved dorsal hippocampal 5-HT and β-adrenergic signaling in female rats, while only 5-HT signaling has been implicated in males (Kohtz and Aston-Jones, 2017). Ovarian hormones (particularly estradiol) also exert direct effects on additional brain regions, including the dorsal striatum, hypothalamus, amygdala, and prelimbic PFC (Dos Anjos Rosário et al., 2022).

**Table 4:** Cocaine-induced changes in neurotransmitter systems in preclinical studies.

**Neurotransmitter systems**	**Brain structures**	**Male**	**Female**	**Reference**	**Additional information**
DA system	Mesolimbic system	Gene regulation		Walker et al., 2022b	Acute (but not chronic) cocaine
	NAcc		Higher SER release	Shimamoto et al., 2011	Acute cocaine
	Dorsal hippocampus			Kohtz and Aston-Jones, 2017	Drug seeking during the initial abstinence
NA system	Dorsal hippocampus			Koht and Aston-Jones, 2017	Drug seeking during the initial abstinence
Glutamate system and Creb pathway	NAcc		GluA1, GluA2	Castro-Zavala et al., 2021	After chronic cocaine
		CREB		Castro-Zavala et al., 2021	After chronic cocaine
	VTA		GluA1, GluA2	Castro-Zavala et al., 2021	After chronic cocaine
		CREB		Castro-Zavala et al., 2021	After chronic cocaine
	mPFC		GluA1, GluA2	Castro-Zavala et al., 2020	After chronic cocaine
		Gria2		Castro-Zavala et al., 2020	After chronic cocaine

NAcc, nucleus accumbens; VTA, ventral tegmental area; mPFC, medial prefrontal cortex; SER, serotonin; Gria2, glutamate receptor ionotropic AMPA subunit 2; GluA1 and GluA2, glutamate receptor AMPA subunits 1 and 2; CREB, cyclic adenosine monophosphate (cAMP) response element-binding. Pink, higher in females; blue, higher in males; grey: no sex differences.

Sex differences in the role of the glutamatergic system in the effects of cocaine have also been reported (for a review, see Giacometti and Barker, 2020). Compared to males, female mice have increased levels of glutamate AMPA receptors GluA1 and GluA2 in the NAcc and VTA, respectively, and decreased levels of CREB (cAMP response element-binding) in these structures (Castro-Zavala et al., 2021). Additionally, higher levels of glutamate AMPA receptors (GluA1 and GluA2) and lower levels of Gria2 (glutamate receptor ionotropic AMPA subunit 2) in the mPFC have been observed (Castro-Zavala et al., 2020). Higher levels of GluA1 resulted in a more rapid and robust cocaine-induced long-term potentiation, which may explain why female mice exhibit faster acquisition of cocaine SA than males (Castro-Zavala et al., 2021). Extinction of cocaine SA and cue-induced reinstatement of cocaine SA induce sex-dependent changes in the expression of genes related to glutamate N-methyl-D-aspartate (NMDA) receptors. In this context, males have shown changes in brain-derived neurotrophic factor-IV, Grin1, Grin2a, and Grin2b throughout the entire phase of withdrawal, while females displayed a modest increase in Grin1 expression only at intermediate withdrawal. These findings suggest that cocaine craving is similarly expressed in males and females, although the time course for its incubation appears to be accelerated in females. Furthermore, the molecular mechanisms underlying craving seem to differ between males and females (Towers et al., 2023b). In addition, the facilitating effects of estradiol on cocaine SA and sensitization are known to require the release of glutamate and the activation of metabotropic glutamate receptors (mGluR5; for review see Dos Anjos Rosário et al., 2022; Nicolas et al., 2022; Peart et al., 2022). Therefore, the increased vulnerability to cocaine observed among females may be explained by sex differences in DA, serotonin, and glutamate mesocorticolimbic neurotransmission.

##### 4.1.2.2 Hormonal influences on cocaine vulnerability in preclinical studies

As in humans, estrogen levels are considered one of the main causes of sex differences in cocaine vulnerability in animal models (Knouse and Briand, 2021; Peart et al., 2022; Torres, 2022). In female rodents, ovariectomy reduced the preference for cocaine over food (Kerstetter et al., 2012), cocaine SA (Lynch et al., 2001; Perry et al., 2013; Ramôa et al., 2013), and cocaine CPP (Satta et al., 2018), while estradiol administration increased cocaine SA (Lynch et al., 2001; Perry et al., 2013) and cocaine CPP (Satta et al., 2018). Additionally, females in the estrus phase (with higher estrogen levels) exhibited stronger responses to the rewarding effects of cocaine (Calipari et al., 2017) and were more susceptible to cue- (Corbett et al., 2021; Nicolas et al., 2019) and priming-induced (Kippin et al., 2005) reinstatement compared to females in the diestrus phase (for a review, see Ahmed et al., 2024, and Torres, 2022). In addition, female rats were found to be more sensitive than males to cocaine-induced locomotion and behavioral sensitization, with estradiol facilitating these sex differences, while progesterone and allopregnanolone attenuated the effects of cocaine (Milivojevic et al., 2016; for a review, see Peart et al., 2022). Estrogen has also been implicated in extinction learning, as ovariectomy has been shown to impede the extinction of cocaine CPP (Twining et al., 2013). Interactions between estrogen receptors and histone deacetylases (enzymes that regulate gene expression) may also contribute to the observed sex differences in the effects of cocaine (Torres, 2022).

### 4.2 Sex differences in response to stress

The hypothalamic–pituitary–adrenal (HPA) axis is a hormonal system that plays a critical role in the stress response. The hypothalamus secretes corticotrophin-releasing hormone (also known as CRF), which stimulates the pituitary gland to produce adrenocorticotropic hormone (ACTH). ACTH enters the bloodstream and triggers the release of hormones from the adrenal glands, primarily cortisol in humans and corticosterone in rodents. These corticoids then inhibit the further release of CRF and ACTH through negative feedback (Habib et al., 2001). Women have lower baseline ACTH (Waldrop et al., 2010) but a higher cortisol response to CRF administration than men (Brady et al., 2009). To induce psychosocial stress in humans, research studies frequently use the Trier Social Stress Task, which requires participants to give an interview-style presentation and take a mental arithmetic test in front of a panel that does not provide feedback (Allen et al., 2016). Other studies have induced stress by exposing individuals to psychological or physical stressors (for example, mental arithmetic or cold pressor tasks), presenting stress imagery, or administering yohimbine.

Sex differences in the response to stress may influence the increased vulnerability of females to developing a CUD. Chronic cocaine use impairs the ability to release cortisol in response to stress (Heesch et al., 1995), and women with a CUD exhibit a more significant decrease in cortisol levels in response to the Trier Social Stress Task than men (Baker et al., 2022; Waldrop et al., 2010). Neuroimaging studies have also demonstrated sex differences in the sensitivity of brain regions responsible for regulating the response to CRF.

Preclinical studies have also demonstrated sex differences in the response to stress. Compared to males, female rodents have more glucocorticoid receptors in the CA1 region of the hippocampus (Kikusui et al., 2005) and exhibit higher levels of corticosterone at baseline and in response to stress, as well as greater sensitivity to CRF and noradrenergic systems, primarily in the locus coeruleus. In contrast, CRF activates the central amygdala and basal nucleus of the stria terminalis to a greater extent in males than in females (see Martin et al., 2021; McRae-Clark et al., 2017, for a review). Social isolation during adolescence induces more transcriptional changes in the brain reward system of male mice (Walker et al., 2022b), evokes a greater HPA axis response to acute footshock in male rats, induces depression-like behaviors, and increases hippocampal brain-derived neurotrophic factor only in male rats (Pisu et al., 2016). In contrast, social isolation in adulthood leads to increased corticosterone levels in female rats (Benite-Ribeiro et al., 2014) and decreases corticosterone levels in males (Brown and Grunberg, 1995).

Thus, there is evidence to suggest that exposure to stress more readily predisposes female rodents to cocaine dependence; however, the increased sensitivity of females to cocaine depends on several variables, including the developmental phase at the time of stress exposure, the stress protocol employed, and the behavioral outcomes evaluated.

## 5 Effects of stress exposure at different developmental stages on the vulnerability to cocaine

### 5.1 Clinical studies

Disruption of the HPA axis and altered extra-hypothalamic CRF-mediated stress responses may contribute to the dysphoria and negative affect that perpetuate drug use and lead to relapse in individuals with CUD. In these individuals, subjective measures of stress and craving in response to the Trier Social Stress task correlated positively (Baker et al., 2022). However, these measures did not correlate with their cortisol response, suggesting a weak association between distress and the HPA axis response during stress (Sinha et al., 2003). Regarding sex differences, clinical research has reported greater sensitivity to CRF and noradrenergic stimulation, as well as an increased neural response to stressful stimuli, in cocaine-dependent women compared to men (Brady et al., 2009; McRae-Clark et al., 2017).

The occurrence of childhood traumatic events increases the likelihood of cocaine relapse and escalated drug use after initial relapse in women, but not in men (Hyman et al., 2008). Cocaine-dependent women also show increased reactivity to psychological and physical stress tasks (Back et al., 2005). While women and men with CUD exhibit similar correlations between subjective stress and craving in response to the Trier Social Stress Test, women with CUD display heightened levels of subjective stress and craving (Baker et al., 2022). A recent review study concluded that women are more sensitive to craving induced by physical (yohimbine) and psychosocial (Trier Social Stress Test) stress, but only during the early stages of abstinence (Nicolas et al., 2022). No sex differences in cocaine craving were observed when stress imagery was presented to abstinent cocaine users, although brain activation in several corticostriatal and frontolimbic areas (dorsomedial and dorsolateral PFC, inferior frontal cortex, cingulate cortex, and insula) was higher in women with CUD (Li et al., 2005; Potenza et al., 2012). Additionally, cocaine-dependent women have been shown to exhibit greater anxiety and craving after exposure to yohimbine plus drug-paired cocaine cues compared to cocaine-dependent men (Moran-Santa Maria et al., 2014). However, cocaine-dependent men demonstrate greater reactivity to drug cue imagery and exhibit increased hyperactivity in the corticostriatal-limbic circuits compared to cocaine-dependent women (Potenza et al., 2012). Reactivity to cocaine cues has been related to daily hassle sensitivity in cocaine-dependent women, but not in cocaine-dependent men (Waldrop et al., 2007). These results suggest that stress increases the salience of cocaine cues for women, supporting the existence of sex differences in vulnerability to craving and relapse under stressful conditions. In addition, after exposure to drug-paired cues (alone or with yohimbine), women (but not men) with CUD showed deficits in sustained attention, evidenced by an increased number of omission errors and longer mean response times. Notably, these differences were specific to individuals with CUD, as they were not observed in individuals without this disorder (Moran-Santa Maria et al., 2016).

Crack cocaine-dependent women who had suffered early-life stress showed more cognitive impairments in executive functions and working memory (Viola et al., 2013), increased levels of proinflammatory cytokines (Levandowski et al., 2014, 2016a), and more alterations in neurotrophic factors (Viola et al., 2014) than those who had not. In addition, crack cocaine-dependent women with and without a childhood trauma history exhibited shorter telomere lengths (indicative of cellular aging) than healthy elderly women, despite being younger. Moreover, among dependent women, those with childhood trauma had significantly shorter telomeres (Levandowski et al., 2016b). A recent study reported higher scores of childhood trauma and increased neutrophil activation and peripheral inflammation in crack cocaine-dependent women compared to healthy counterparts, including higher levels of plasma cytokines, increased neutrophil phagocytosis and production of neutrophil extracellular traps, elevated levels of intracellular reactive oxygen species, and more activated phosphorylated protein kinase B and mitogen-activated protein kinases (MAPK) (Funchal et al., 2024). It would be interesting to determine if similar results are observed in men with crack dependence. In addition, it is crucial to study the role of the immune system in the effects of stress exposure during critical developmental stages in both men and women with CUD.

### 5.2 Preclinical studies

#### 5.2.1 Prenatal stress

Only three studies have examined the effects of prenatal stress on the vulnerability of female rodents to cocaine (see Table 5). The research in question exposed pregnant rats to restraint, and the behavior of their offspring was evaluated in adulthood; in particular, the reinforcing effects (Bagley et al., 2019; Thomas and Becker, 2019; Thomas et al., 2009), psychomotor-sensitizing effects (Bagley et al., 2019; Thomas et al., 2009), and conditioned rewarding effects of cocaine (Bagley et al., 2019), as well as vulnerability to addiction-like behavior (Thomas and Becker, 2019). These studies compared prenatally stressed male and female rats with non-stressed control rats. In the initial study by Thomas et al. (2009), rats were trained in cocaine SA at two constant doses (0.2 and 0.5 mg/kg/infusion) or with an escalating-dose regimen. At the lower constant dose (0.2 mg/kg/infusion), prenatally stressed rats tended to acquire cocaine SA faster than control animals, and no sex differences in the acquisition of SA were observed (although the overall intake of cocaine per session was higher among males than females). At the higher constant dose (0.5 mg/kg/infusion), prenatally stressed males showed higher cocaine intake and acquired cocaine SA in fewer sessions than control males, but no effects of prenatal stress were observed in females. Similarly, with the escalating-dose regimen (starting at 0.3 mg/kg/infusion and progressively increasing the dose across sessions), prenatal stress only affected male rats. A higher percentage of these males acquired cocaine SA within three sessions, leading to a shorter time to meet acquisition criteria and a greater cocaine intake compared to control male rats.

**Table 5:** Sex differences in the influence of stress on the behavioral effects of cocaine in rodents.

**Developmental period**	**Stress protocol**	**Timing of stress exposure**	**Behavioral outcome**	**Male**	**Female**	**References**	**Additional information**
Prenatal	Maternal restrain	Before cocaine exposure	Behavioral sensitization			Thomas et al., 2009	
			SA			Thomas et al., 2009	
			Behavioral sensitization			Bagley et al., 2019	Attenuation in males and few effects in females
			CPP			Bagley et al., 2019	
			Motivation for cocaine SA			Thomas and Becker, 2019	Extended SA protocol
Neonatal	Isolation	Before cocaine exposure	SA			Kosten et al., 2004	
			SA			Kosten et al., 2006	
			CPP			Ganguly et al., 2019	
			Cue-induced reinstatement of SA			Lynch et al., 2005	
	Maternal separation	Before cocaine exposure	SA			Matthews et al., 1999	No sex differences in adolescence
			Behavioral sensitization			Kikusui et al., 2005	
	Maternal separation with early weaning	Before cocaine exposure	SA			Castro-Zavala et al., 2020	Although without stress females showed higher cocaine SA
			SA			Castro-Zavala et al., 2021	
	Episode of maternal separation	Before cocaine exposure	Negative affect during cocaine abstinence			Bis-Humbert et al., 2021b; Bis-Humbert and García-Fuster, 2021	Separate studies for each sex; emerged in adolescence (males) or adulthood (females)
	Repeated cross fostering	Before cocaine exposure	CPP			Di Segni et al., 2019	But induced anhedonic-like effects in males
Adolescent	Stresssors combination	No cocaine exposure	Addiction traits			Hynes et al., 2018	Separate studies for each sex
			Depression-like symptoms			Bourke and Neigh, 2011	
		Before cocaine exposure	Impulsivity			Paine et al., 2021	
		Before cocaine exposure	Hiperactivity			Lepsch et al., 2005; Rowson et al., 2018	
	Isolation	Before/during cocaine exposure	Motivation for cocaine SA			Fosnocht et al., 2019	Although males were more sensitive in a FR1 schedule
		Before cocaine exposure	CPP			Walker et al., 2022a	Increased in males but decreased in females
		Before/during cocaine exposure	SA			Westenbroek et al., 2013	Late adolescent
	Isolation		Depression-like symptoms			Pisu et al., 2016	No cocaine exposure
	Social defeat	Before cocaine exposure	SA			Haney et al., 1995	Escalation and binges
			SA			Holly et al., 2012	
			Behavioral sensitization			Shimamoto et al., 2018	
		Before cocaine exposure	Cue-induced seeking			Morales-Silva et al., 2024	After 30 days of forced abstinence of cocaine SA
			Priming-induced seeking			Morales-Silva et al., 2024	
Adulthood	Footshock	After cocaine SA	SA, priming- and cue- induced seeking			Farrell et al., 2019	No sex differences in punishment-induced suppression of SA
			Responses in time-out SA			Gaulden et al., 2025	
		After extinction of cocaine SA	Stress-induced reinstatement of SA			Connelly et al., 2020	
			Priming-induced reinstatement SA			Doncheck et al., 2020	
	CRF	After extinction of cocaine SA	Stress-induced reinstatement of SA			Buffalari et al., 2012	
	Yohimbine	After extinction of cocaine SA	Stress-induced reinstatement of SA			Anker and Carroll, 2010	Higher with more estradiol and lower with more progestins
			Stress-induced reinstatement of SA			Zlebnik et al., 2014	
			Stress-induced reinstatement of SA			Zhou et al., 2012	
			Cue-induced reinstatement of SA			Feltenstein et al., 2011	
	Restrain	After extinction of cocaine SA	Stress-induced reinstatement of SA			Hamilton et al., 2018	higher in females during diestrus and proestrus phases
			Priming-induced reinstatement SA			Doncheck et al., 2020	
		After cocaine SA	Cue-induced cocaine seeking			Edson and Ball, 2022	Punishment-induced abstinence

CRF, corticotropin-releasing factor; SA, self-administration of cocaine; CPP, conditioned place preference induced by cocaine. Pink indicates that the effect was greater in females than in males; blue indicates that the effect was stronger in males than in females; gray indicates no clear sex differences.

Conversely to what was observed in SA, prenatal stress only enhanced psychomotor sensitization in females. Prenatal stress did not affect the acute motor effects of cocaine in male or female rats; however, after repeated exposure to cocaine, prenatally stressed females exhibited an increase in the psychomotor-activating effects of cocaine compared to non-stressed females. Prenatal stress did not affect cocaine behavioral sensitization in male rats, and females overall developed more robust locomotor sensitization than males. Thomas et al. (2009) suggested that sex differences interact with prenatal stress to modulate the sensitivity of rats to the reinforcing and behavioral-sensitizing effects of cocaine. However, the authors speculated that the lack of effects of prenatal stress on cocaine SA in females might have been due to a ceiling effect, since control females nearly achieved the maximum acquisition rate. They hypothesized that the effects of prenatal stress in female rats only emerge after chronic drug exposure. In a more recent study (Thomas and Becker, 2019), male and female rats exposed to prenatal stress were trained using an extended long-access cocaine SA protocol (0.8 mg/kg/infusion, once daily, five consecutive days per week, for seven weeks) to evaluate the presence of addiction-like traits, such as higher motivation for cocaine and persistence of drug-seeking in the absence of reinforcement. SA training included a combination of FR and PR schedules of reinforcement, and the rats only returned to their home cages after reaching the established criterion. The first session began with FR1 and a criterion of five infusions, which increased over nine sessions. The maintenance period consisted of FR5 sessions, three signaled drug sessions (with a maximum of nine infusions), and no-drug periods (chamber lights out and no infusions) between drug sessions. Motivation for cocaine was determined by the breakpoint score in PR sessions before and after the maintenance phase. The persistence of drug-seeking behavior in the absence of reinforcement was measured by the number of nose pokes in the active hole during “no drug” periods. Regardless of their stress history, females self-administered cocaine at a faster rate than males. In addition, stressed rats of both sexes exhibited more addiction-like criteria than controls, though this effect was more pronounced in females. The breakpoint scores of the stressed female group shifted upward relative to the other groups, and a greater percentage of females with prenatal stress scored at or above the overall median breakpoint value. These results suggest that motivation for cocaine was higher in prenatally stressed females. In addition, a higher percentage of females with prenatal stress met the criterion “Drug seeking in the absence of reinforcement” than subjects from any other group (Thomas and Becker, 2019). These results suggest that stressed females are more vulnerable to developing addiction-like behavior after chronic cocaine exposure.

Genotype is also an important variable that modulates the effects of prenatal stress in both sexes. A study by Bagley et al. (2019) showed that strain, prenatal stress, and sex interact to modulate the locomotor effects of cocaine, behavioral sensitization, and cocaine-induced CPP. Sex differences in the effects of prenatal stress varied across the strains; most displayed attenuation of cocaine-induced sensitization and CPP when males were stressed. Again, prenatal stress in females of most strains did not affect sensitization, and only a slight effect was observed in CPP. These results suggest that the effects of prenatal stress depend on sex and strain. There are likely genetic variants that interact with prenatal stress to increase or decrease sensitivity to cocaine, although males of many strains demonstrate higher sensitivity to the effects of prenatal stress (Bagley et al., 2019).

In sum, prenatal stress induced by maternal restraint was shown to affect female rats more severely than male rats, increasing cocaine behavioral sensitization (Thomas et al., 2009) and SA (Thomas and Becker, 2019). However, in another study, prenatally stressed male rats were more sensitive to cocaine SA than female rats (Thomas et al., 2009). Further research is needed to examine sex differences in the effects of prenatal stress on cocaine vulnerability, since only one protocol involving gestational stress in rats has been used thus far. The evidence, though limited, suggests that female rats are more sensitive to cocaine with extended protocols of SA, which better capture addiction-like behaviors.

#### 5.2.2 Early-life stress

Neonatal isolation stress (PND 2-9) does not alter baseline DA levels, but it enhances cocaine-induced increases in DA (Kosten et al., 2003) and facilitates the acquisition of cocaine SA (with FR1, FR3, and PR schedules of reinforcement) in adult rats of both sexes, although the latter effect is more evident in females (Kosten et al., 2004, 2006). This stress protocol was also found to potentiate cocaine-seeking behavior (cue-induced reinstatement) after 10 days of forced abstinence in adult male and female rats, without sex differences (Lynch et al., 2005). Extending neonatal isolation until weaning (from PND 10–20) increases the CPP induced by cocaine during adolescence, but only in male rats (Ganguly et al., 2019).

Adult rodents exposed to nest separation during early life have shown an increased response to cocaine in adulthood (Kikusui et al., 2005; Matthews et al., 1999). Stress induced by repeated maternal separation increases cocaine-induced hyperactivity in late adolescence, regardless of sex. However, in adulthood, only male mice with a history of maternal separation exhibit cocaine-induced behavioral sensitization (Kikusui et al., 2005). Repeated episodes of maternal separation (between PND 5–20) also affect the acquisition of cocaine SA, though the effects depend on the dose administered (0.05, 0.08, and 0.5 mg/kg/injection). At the low dose, stressed animals of both sexes showed delayed acquisition of cocaine SA compared to controls, while at the intermediate dose, stressed females demonstrated facilitated acquisition. After stable SA was established, the dose-effect function shifted to the right for stressed females and downward for stressed males compared to controls (although the lighter body weights of the females meant that they received a higher unit dose per unit body weight than the males). At the dose that elicited maximal responding (0.03 mg/injection), stressed males tended to self-administer less cocaine than controls and executed fewer lever responses overall at each tested dose; conversely, stressed females self-administered significantly more cocaine than controls (Matthews et al., 1999). These results indicate that maternal separation can induce contrasting sex-dependent effects on cocaine SA, which also depend on the cocaine dose used.

Other studies have examined the effects of maternal separation with early weaning (MSEW) on motivation to seek cocaine in a 7-day SA protocol and its association with despair-like behavior and changes in the glutamatergic system in adult mice. A study by Castro-Zavala et al. (2020) reported that this early-life stress protocol induced effects only in male mice, which showed increased acquisition of cocaine SA and changes in glutamate molecules (an increase of GluA1 in the NAcc and a decrease of the GluA1/GluA2 ratio in the VTA). However, the absence of stress effects in female mice may be due to significant sex differences in cocaine SA. Female mice acquire SA faster, consume more cocaine, and display a higher percentage of acquisition than males. In addition, basal differences in glutamatergic AMPA receptors have been observed between drug-naïve male and female mice, which may underlie the higher motivation of females to self-administer cocaine. These differences include higher levels of GluA1 in the NAcc and GluA2 in the VTA, as well as lower levels of CREB in both the NAcc and VTA (Castro-Zavala et al., 2020). In a subsequent study, the same authors replicated the results of SA and extended the evaluation of glutamatergic receptors to the PFC. Control female mice exhibited a higher motivation to seek cocaine and had higher levels of GluA1 and GluA2 in the medial PFC than males. In addition, maternal separation with early weaning increased motivation for cocaine-seeking only in males, who also showed an increase in Gria2 and a decrease in the Gria1/Gria2 ratio in the mPFC. The same study also demonstrated an association between despair-like behavior and motivation for cocaine SA; females displayed higher immobility in the tail suspension test compared to males, and early-life stress increased immobility in male mice only (Castro-Zavala et al., 2021). In a recent study in which only female mice underwent early weaning (Arenas et al., 2022), it was shown that this stress protocol increased cocaine-seeking behavior in female mice. Specifically, early-stressed adult female mice exhibited decreased cocaine-induced behavioral sensitization and cocaine SA compared to non-stressed controls. However, female mice exposed to early-life stress showed reinstatement of cocaine CPP after cocaine priming, an effect not observed in non-stressed female mice. In addition, this study demonstrated that females with lower prepulse inhibition (a trait associated with several psychiatric disorders) exhibited more anhedonia- and despair-like behaviors and were more sensitive to the negative consequences of MSEW on cocaine-seeking behavior (Arenas et al., 2022). MSEW also induced depressive-like behaviors and changes in sensitivity to the rewarding effects of cocaine in adolescent male mice (Gracia-Rubio et al., 2016), but the effects of MSEW on adolescent females have not yet been evaluated.

Genotype also modulates the influence of early-life stress on the effects of cocaine on catecholamine release and CPP acquisition in female mice. Adult female C57BL6J mice that had experienced repeated cross-fostering displayed increased NA and DA release in the mPFC and NAcc, respectively, following cocaine administration. These mice also exhibited greater vulnerability to cocaine CPP compared to control mice. Conversely, DBA female mice undergoing repeated cross-fostering exhibited reduced release of NA and DA, as well as diminished sensitivity to cocaine (Di Segni et al., 2017). In a subsequent study, the same authors compared the effects of repeated cross-fostering in male and female C57BL6J mice (Di Segni et al., 2019). This stress protocol increased the rewarding effects of cocaine in CPP in females but did not affect cocaine CPP in males. Conversely, repeated cross-fostering induced anhedonic-like effects in males, such as increased immobility in the forced swimming test, but not in females. As the authors indicated, these results suggest that early-life stress produces impairments in both sexes in ways that manifest in adulthood as opposite phenotypes: anhedonia-like in males and addiction-like in females (Di Segni et al., 2019).

The effects of a single episode of maternal separation for 24 h from PND 9 to PND 10 on subsequent vulnerability to the negative affect induced by cocaine withdrawal have been studied in male (Bis-Humbert et al., 2021b) and female (Bis-Humbert and García-Fuster, 2021) rats. Rats exposed to a single episode of maternal deprivation demonstrated heightened negative affect following the administration of cocaine during adolescence or adulthood, depending on sex (Bis-Humbert and García-Fuster, 2021; Bis-Humbert et al., 2021a,b). An increase in negative affect during cocaine abstinence has been linked to a higher vulnerability to developing addictive-like responses, such as persistence in drug seeking and higher relapse rates. Changes in affective-like behavior were evaluated using the open field, forced swim, novelty-suppressed feeding, and sucrose preference tests. In males, maternal deprivation or 7 days of cocaine treatment during adolescence increased negative affect in adulthood (but not during adolescence). The combination of early-life stress and adolescent cocaine treatment increased negative affect during adolescence, while maternal deprivation and/or cocaine treatment during adulthood did not induce behavioral changes (Bis-Humbert et al., 2021b). In contrast, in females, the combination of early-life stress and adolescent cocaine did not induce behavioral effects during adolescence, but negative affect emerged during adulthood in the form of anxiogenic- and depressive-like effects, as indicated by decreased exploration in the open field test and increased immobility in the forced swim test, respectively. These effects mainly occurred during abstinence after cocaine re-exposure in adulthood (Bis-Humbert and García-Fuster, 2021). Since these studies used the same methodology, they allow for comparison between males and females exposed to maternal deprivation and cocaine. In this sense, there are sex differences in when vulnerability to the negative impact on affect emerges: during the adolescent period for males and in adulthood for females. In fact, females showed resilience during adolescence and required drug re-exposure in adulthood to display increases in negative affect.

In summary, neonatal isolation stress enhances the ability of cocaine to increase DA levels in pups of both sexes (Kosten et al., 2003), while this stressful experience (Kosten et al., 2004, 2006) and neonatal maternal separation (Matthews et al., 1999) both increase cocaine SA, particularly in females. In contrast, neonatal isolation increases cocaine-induced CPP only in male rats (Ganguly et al., 2019) and facilitates cue-induced reinstatement of cocaine SA in both sexes (Lynch et al., 2005). In mice, maternal separation increases behavioral sensitization (Kikusui et al., 2005) and the acquisition of cocaine SA (Castro-Zavala et al., 2020, 2021) only in males, while repeated cross-fostering increases cocaine CPP only in females (Di Segni et al., 2019). While female rats exposed to stress in early life were more vulnerable to cocaine SA than males, this was not true for other behavioral outcomes, such as CPP or behavioral sensitization. In addition, results obtained in mice were controversial. Therefore, it is difficult to establish a clear conclusion, as the studies employed different methodologies, with various stress protocols, species, strains, and evaluations of short- or long-term effects.

#### 5.2.3 Stress during adolescence

The effects of exposure to stress during adolescence on subsequent vulnerability to cocaine have been studied mainly through the social isolation protocol. This well-established preclinical model of addiction susceptibility involves housing animals individually from the beginning of adolescence for several days or until adulthood. Social isolation from PND 21 was shown to increase the response to cocaine SA (FR1) only in adult male mice. However, no sex differences were observed in the enhanced vulnerability to addiction-like behaviors induced by social isolation, including greater motivation for cocaine self-administration, as evidenced by a higher breaking point, cocaine seeking during cue-induced reinstatement, and cocaine-induced neuronal activation within the NAcc core and shell, ventral pallidum, dorsal bed nucleus of the stria terminalis, lateral septum, and basolateral amygdala (Fosnocht et al., 2019). Similarly, no sex differences were reported in the biochemical effects of social isolation, including decreased presynaptic glutamate transmission at synapses from the ventral hippocampus to the NAcc core (Deutschmann et al., 2022). Social isolation for 10 days (PND 22-42) produced opposite effects on cocaine CPP, increasing CPP in male mice and decreasing it in females (Walker et al., 2022a). This stress protocol also reduced sex differences in anxiety-like behaviors. In addition, isolated males displayed a robust transcriptional response to cocaine (cfos) in the medial amygdala, and social isolation induced a loss of sex differences in gene expression in this structure (Walker et al., 2022a). Adolescent social isolation reduced sex differences in baseline gene expression in the PFC, NAcc, and VTA but increased sex differences in response to cocaine in these structures due to changes in isolated males (Walker et al., 2022b). The authors of these studies stated that “social isolation induces gene expression profiles in males that more closely resemble those of group-housed females, suggesting that social isolation ‘feminizes' the male transcriptome” (Walker et al., 2022b). Social isolation beginning in late adolescence (PND 42) was also found to increase motivation to self-administer cocaine, particularly in female rats (Westenbroek et al., 2013).

Recently, the vicarious intermittent social defeat protocol has been used to evaluate the effects of adolescent social stress exposure on cocaine vulnerability in female mice. After witnessing the social defeat of a male conspecific mouse during late adolescence (PND 47–56), adolescent females exhibited increased corticosterone levels and displayed anxiety- and depression-like behaviors (Martínez-Caballero et al., 2024; Ródenas-González et al., 2023) and heightened vulnerability to cocaine reward in adulthood, as indicated by the development of CPP after conditioning with an ineffective dose of cocaine (Ródenas-González et al., 2023). The behavioral profile of female mice shortly after their exposure to vicarious defeat influenced their subsequent vulnerability or resilience to cocaine CPP in adulthood. This suggests that not all female mice exposed to stress during early (PND 27–36) (Martínez-Caballero et al., 2025) or late adolescence (Martínez-Caballero et al., 2024) develop enhanced vulnerability to cocaine. Thus, exposure to vicarious social defeat increases the rewarding effects of cocaine only in vulnerable female mice (Martínez-Caballero et al., 2024, 2025). Similar results have been observed in studies with male mice exposed to intermittent social defeat protocols during early or late adolescence (Calpe-López et al., 2020, 2023).

Finally, a combination of social isolation, food restriction, forced swim, restraint, and exposure to predator odor (on PND 21–35) was reported to increase addiction-risk traits in adulthood, including an enhanced preference for novelty and a greater attribution of incentive value to reward cues, effects that were more evident in female rats (Hynes et al., 2018). Juvenile stress induced by exposure to various stressful events (social isolation, swim stress, restraint, footshock, and constant lights) on PND 25–29 increased the impulsivity induced by cocaine in female rats, but not in males, an effect that might reflect enhanced susceptibility to addiction (Paine et al., 2021). Similarly, adolescent stress induced by a combination of stressful conditions (isolation, restraint, and social defeat on PND 37–49) produced more behavioral alterations (decreased sucrose consumption, hyperactivity in the elevated plus maze, decreased activity in the forced swim test, and a blunted corticosterone response to acute forced swim stress) in female rats than in male rats, both at the end of adolescence and during adulthood (Bourke and Neigh, 2011). On the other hand, exposure to this combination of stressors during adolescence was found not to affect the hyperactivity or behavioral sensitization induced by cocaine in adolescent or adult female rats (Rowson et al., 2018). However, it had previously been reported that exposure to adolescent stress (restraint or a combination of stressors) increased cocaine-induced locomotor activity in male rats (Lepsch et al., 2005).

It is important to note the limited number of studies that have evaluated the role of stress during adolescence on the effects of cocaine, especially considering that adolescence is a vulnerable period of development. Many studies have used social isolation to induce stress in mice; however, other protocols, such as social defeat, could have more ethological and ecological validity as a model of bullying. Studies evaluating the effects of vicarious social defeat on cocaine reward have only been performed in female mice. It would be of interest to study males to detect sex differences in the effects of this kind of stress. Using both early- and late-adolescent animals could also contribute to a better understanding of the role of stress in cocaine vulnerability. Thus far, the results have not clearly demonstrated that stressed females during adolescence are more responsive to cocaine than males.

#### 5.2.4 Stress during adulthood

Several protocols have been used to evaluate the effects of stress experienced in adulthood—including chronic footshock, chronic social defeat, acute and chronic restraint, and acute administration of CRF or yohimbine—on vulnerability to the acquisition of cocaine SA, behavioral sensitization induced by cocaine, and reinstatement of cocaine-seeking behavior.

In rats that were stressed with chronic footshocks before acquiring SA, sex differences emerged after 30 days of forced abstinence. Stressed female rats exhibited higher cue-induced seeking than stressed male rats and control female rats, while stressed male rats exhibited higher priming-induced seeking than male control rats (Morales-Silva et al., 2024). Conversely, no sex differences have been found in punishment-induced suppression of SA or in cue- and priming-induced seeking after voluntary, punishment-induced abstinence (Farrell et al., 2019). Administering footshocks during the acquisition phase was reported to increase cocaine SA similarly in male and female rats. In non-stressed control animals, however, males exhibited more time-out responses (defined as responses in the time immediately following a cocaine infusion when responding to the active lever is not reinforced) than females. Both stressed males and females displayed more time-out responses overall than the non-stressed groups did across SA1–14. However, stressed females presented greater total infusions during the first 30 min of SA sessions (SA sessions 1–14) and greater time-out responses during SA14 than stressed males (Gaulden et al., 2025).

Exposure to social defeat stress in adulthood increases cocaine SA in both sexes (Haney et al., 1995). In females, the experience of episodic social defeat by a lactating female increases the SA of cocaine in defeated rats compared to non-defeated control females (Haney et al., 1995). Although social defeat has been shown to result in behavioral and dopaminergic cross-sensitization in both sexes, the effects are larger and longer-lasting in stressed females, who are also more vulnerable to the escalation of cocaine intake induced by social defeat stress (Holly et al., 2012). For instance, stress increased binge duration in both sexes; however, defeated females binged for longer than defeated males. Additionally, cocaine-induced DA release in the NAcc was evident for only 40 min in males and at least 120 min in females. These alterations in DA may contribute to the more dysregulated cocaine use observed in stressed female rats. Furthermore, this study suggests that estrogens play a facilitatory role in behavioral sensitization in both control and defeated female rats (Holly et al., 2012).

Chronic social defeat stress, which involves daily exposure to episodes of defeat by a lactating dam for 21 days, induced physiological alterations and behavioral deficits indicative of depression-like behavior in defeated female rats compared to non-defeated control females. These alterations included reduced weight gain, disruption of estrous cycles, blunted release of DA and serotonin in NAcc in response to cocaine, increased immobility, and reduced preference for saccharin (Shimamoto et al., 2011). As mentioned previously, not all animals exposed to chronic social defeat stress are equally affected. Some female rats are susceptible to the effects of chronic defeat, as evidenced by a decline in saccharin intake, which has been linked to subsequent cocaine-taking behavior (Shimamoto et al., 2011, 2015). In the experiment by Shimamoto et al. (2015), two subgroups of defeated female rats were observed with respect to their saccharin intake: stress-sensitive rats showed lower saccharin intake after defeat, while stress-resistant rats displayed a level of saccharin intake similar to that of non-defeated rats. Chronic social defeat induced different biochemical changes in the NAcc shell in stress-sensitive and stress-resistant rats: increased serotonin in the former and reduced DA in the latter. Social defeat did not alter cocaine SA in the FR or PR schedules; however, in a 24-h cocaine binge session, stress-sensitive and stress-resistant rats presented lower and higher cocaine infusions, respectively, compared to the non-defeated control group. Similarly, after a cocaine challenge, dopamine levels increased in non-defeated and stress-sensitive rats, but not in stress-resistant rats, while serotonin levels were higher in stress-sensitive rats than in stress-resistant rats and non-stressed controls (Shimamoto et al., 2015). Furthermore, chronic social defeat stress has been reported to induce sex-dependent effects on cocaine-induced behavioral sensitization in rats. Defeated females showed increased behavioral sensitization and decreased levels of the glutamate transporter in the NAc and PFC, while defeated males exhibited the opposite behavioral and neurochemical changes (Shimamoto et al., 2018).

Exposure to stress also affects the reinstatement of cocaine-seeking behavior, with some sex-related differences. For instance, stress-induced reinstatement of cocaine seeking is stronger in female than in male rodents. The administration of CRF (Buffalari et al., 2012) and yohimbine (Anker and Carroll, 2010; Zhou et al., 2012; Zlebnik et al., 2014) reinstated cocaine SA in rats of both sexes, but female rats exhibited greater stress-induced reinstatement in response to yohimbine than males, and allopregnanolone (a progesterone metabolite) blocked this effect in females but not in males (Anker and Carroll, 2010). Similarly, Buffalari et al. (2012) reported that the reinstatement effect of CRF was highly variable, affecting only a subpopulation of rats, and that a greater number of female rats were highly sensitive to CRF compared with males. Additionally, female rats showed greater stress-induced reinstatement than males after footshock administration (Connelly et al., 2020). Other studies have failed to observe sex differences in yohimbine-induced reinstatement of cocaine SA (Zhou et al., 2012; Zlebnik et al., 2014) or restraint-induced reinstatement of cocaine CPP (Hamilton et al., 2018).

No sex differences were observed in restraint potentiation of priming-induced reinstatement (Doncheck et al., 2020), but intermittent footshock potentiated priming-induced reinstatement in males and not in females (Doncheck et al., 2020). However, the effects of stress (restraint) exposure and corticosterone administration on priming-induced reinstatement of cocaine-seeking behavior in females have been shown to be greater during the diestrus and proestrus phases (Doncheck et al., 2020), with higher reinstatement in stressed females with elevated levels of estradiol (Doncheck et al., 2020).

Finally, chronic restrained stress for seven consecutive days (3 h per day) during the punishment-induced extinction of cocaine SA, influenced cue-induced seeking depending on sex, with only female mice showing increased vulnerability to the incubation of cocaine craving and potentiation of cue-induced cocaine seeking (Edson and Ball, 2022). Sex differences in punishment-induced suppression of cocaine SA have also been reported, with male rats displaying greater resistance to punishment for cocaine SA with footshocks (Edson and Ball, 2022). However, no sex differences in cocaine SA were observed when punishment was induced with higher shock intensities and in a different context (Farrell et al., 2019). These results suggest that not all stressors are equal in their ability to potentiate drug-seeking behavior and that sex is an important variable that modulates their efficacy. Levels of female gonadal hormones may contribute to stress reactivity and reinstatement of cocaine seeking, as these effects are potentiated by higher levels of estradiol and decreased with higher levels of progestins (Feltenstein et al., 2011).

Many studies have examined the role of stress in cocaine-induced behaviors in adult rodents. In general, females displayed a greater vulnerability to the escalation of cocaine SA and craving, as well as cue- and stress-induced reinstatement of cocaine seeking. Conversely, females were not more vulnerable to priming-induced reinstatement.

## 6 Sex differences in the neurobiological mechanisms underlying the role of stress in cocaine-related behaviors

Several functional magnetic resonance imaging studies have demonstrated sex differences in brain activation during exposure to stress imagery in abstinent cocaine-dependent subjects. Using script-guided imagery of neutral or stressful situations, researchers observed different brain activation patterns between the sexes. During stress imagery, women showed greater activation than men in the left frontolimbic areas (including the anterior cingulate cortex and the insula) and the right posterior cingulate cortex (Li et al., 2005). In both sexes, however, activity in the left anterior cingulate and right posterior cingulate cortices evoked by stress imagery correlated inversely with craving ratings on the Likert scale (Li et al., 2005). Stress imagery can also induce alexithymia, which is relevant, as difficulty recognizing one's own emotions can contribute to relapse into drug use. When processing stress, different brain activation patterns have been observed in male and female cocaine-dependent patients with alexithymia. Women displayed hypoactivation in the left cortical hemisphere and subcortical regions of the right hemisphere, while men showed hypoactivation in the right frontal cortex and putamen (Li and Sinha, 2006). Another study demonstrated that, although non-sex-specific alterations in insular morphometry were observed in regular cocaine users, the association between craving (as measured by the Desire for Drug Questionnaire score) and insular morphometry differed between women and men; specifically, the association between right insular volume and stress-relief craving was positive in women and negative in men (Malek et al., 2022).

Only a few studies have examined sex differences in the neurobiological mechanisms underlying the role of stress in the behavioral effects of cocaine in rodents. Fatty acid-binding proteins, which transport anandamide to the fatty acid amide hydrolase enzyme, may be involved in cocaine seeking under stressful conditions in mice of both sexes. In particular, restraint-induced reinstatement of cocaine CPP and corticosterone release were blocked by deleting fatty acid-binding proteins (FABP 5/7) in both male and female mice (Hamilton et al., 2018). On the other hand, a sex-specific role for tumor necrosis factor (TNF) and AMPA receptors in the effects of stress on cocaine-induced CPP has been demonstrated. Maternal separation increased TNF levels and reduced GluA2 expression in both the PFC and NAcc of adolescent male rats, but not in females; these neurochemical and behavioral effects of maternal separation were reversed by ibudilast and TNF blockade in male mice, but these treatments had no effect on female mice (Ganguly et al., 2019). Additionally, sex-dependent effects of repeated cross-fostering have been observed in cellular-fos (c-fos) expression induced by the forced swim test in the infralimbic cortex, NAcc core, and caudate-putamen; this expression increased in female mice but decreased in male mice (Di Segni et al., 2019). Other studies involving only female mice have shown that the increased sensitivity to cocaine CPP in adult females exposed to repeated cross-fostering was accompanied by cocaine-induced increases in NA and DA release in the mPFC and NAcc, respectively, as well as by morphological changes (spine density) in cortical pyramidal neurons and accumbal medium spiny neurons. Additionally, there were increased cocaine-induced c-fos expression in the prelimbic cortex, NAcc core and shell, and lateral and basolateral amygdala, and decreased c-fos expression in the CA1 region and dentate gyrus compared to non-stressed control female mice (Di Segni et al., 2017, 2019). Furthermore, adult female mice exposed to repeated cross-fostering exhibited reduced Xlr4 expression (a gene involved in chromatin remodeling and dendritic spine morphology) in the NAcc, while mice with down-regulated Xlr4 displayed higher sensitivity to cocaine CPP, reinstatement following withdrawal, increased DA levels in the NAcc, and altered spine density in medium-sized spiny neurons of the NAcc after cocaine administration (Di Segni et al., 2020).

Restraint and administration of CRF potentiated priming-induced reinstatement of cocaine SA in both sexes, and these effects required the mobilization of 2-arachidonoylglycerol in prelimbic interneurons and the activation of cannabinoid CB1 receptors (CB1R) in the prelimbic-PFC pathway, which induced suppression of inhibitory synaptic activity (disinhibition) of layer V pyramidal neurons in male and female rats (Doncheck et al., 2020). In contrast, sex differences have been observed in other brain areas involved in the stress response, such as the dorsal hippocampus, locus coeruleus, and dorsal raphe. Stress induced by non-reinforced drug-seeking behavior during the early stages of abstinence has been associated with NA (locus coeruleus) and 5-HT (dorsal raphe) inputs to the dorsal hippocampus. On the first day of extinction, male and female rats showed increased Fos expression in these structures, and β-adrenergic/5-HT1A/1B and 5-HT1A/1B receptor antagonists decreased drug-seeking behavior on the same day. However, blocking 5-HT1A/1B receptors (but not β1 and β2 receptors) in the dorsal hippocampus was effective in males, while blocking 5-HT1A/1B, β1, and β2 receptors was effective in females; this suggests that cocaine-seeking during early abstinence involves 5-HT signaling in the dorsal hippocampus in males and both 5-HT and β-adrenergic signaling in females (Kohtz and Aston-Jones, 2017).

Finally, other studies have observed sex-specific alterations in the neuronal activity of male and female rodents exposed to stress and cocaine. For instance, female rats exposed to chronic social defeat stress exhibited cocaine-induced hyperactivity associated with changes in glutamate/glutamine transfer, including reduced protein levels of their transporters in the NAcc and PFC; conversely, male rats showed the opposite changes (Shimamoto et al., 2018).

## 7 Sex differences in the pharmacological modulation of the effects of stress on cocaine-induced behaviors

### 7.1 Clinical studies

To date, clinical studies have evaluated three drugs—progesterone, guanfacine, and oxytocin—for their potential to prevent stress-related effects in individuals dependent on cocaine. Progesterone has been shown to play a protective role in stress-induced cocaine craving in women. Cocaine-dependent females with low progesterone levels exhibited less stress-induced craving than those with high progesterone levels (Moran-Santa Maria et al., 2018; Sinha et al., 2007). Consistent with this protective role of progesterone, administering this hormone (400 mg/day for seven consecutive days) decreased corticosterone and craving induced by cue imagery, as well as improved Stroop performance (a measure of inhibitory control) in abstinent, treatment-seeking, cocaine-dependent individuals of both sexes compared to a placebo. Furthermore, women (but not men) treated with progesterone reported beneficial effects following stress imagery, including decreased levels of negative emotions and increased levels of relaxation (Fox et al., 2013). Administering progesterone (400 mg/day for seven consecutive days) to cocaine-dependent individuals increased allopregnanolone plasma levels compared to a placebo, with no sex differences detected. Those with higher allopregnanolone levels displayed decreased cortisol levels and a higher positive mood at baseline. They also had a higher cortisol response to stress imagery and improved Stroop performance after drug-cue and stress imagery. Additionally, they showed reduced cocaine craving after neutral, drug-cue, and stress imagery conditions. These results suggest that the neuroactive steroid allopregnanolone mediates the beneficial effects of progesterone on stress arousal, inhibitory control, and drug craving, producing similar effects in men and women with cocaine dependence (Milivojevic et al., 2016).

The administration of the alpha-2 adrenergic agonist guanfacine (2 or 3 mg) for 3 weeks to early abstinent, treatment-seeking, cocaine-dependent males and females reduced sympathetic tone, stress, systolic blood pressure, and craving induced by nicotine cues in both sexes compared to placebo. However, guanfacine was only effective in attenuating cocaine craving, alcohol craving, anxiety, and negative emotions in females following exposure to drug-associated cues, stress, and combined cues plus stress imagery (Fox et al., 2014). Similarly, guanfacine improved performance on the Stroop task compared to placebo in females only (Milivojevic et al., 2017).

The neuropeptide oxytocin has shown therapeutic potential for reducing stress in individuals with addictive disorders (Che et al., 2021). A functional magnetic resonance imaging study examined how intranasal oxytocin (40 IU) affected brain activation in the right amygdala and dorsomedial PFC when cocaine-associated cues were presented to individuals with cocaine dependence, with or without a history of childhood trauma. Compared to the placebo group, the oxytocin groups exhibited reduced cocaine cue reactivity in the dorsomedial PFC, regardless of sex or childhood trauma history. However, the effects of oxytocin on amygdala cue reactivity were dependent on sex and trauma history. Oxytocin reduced the amygdala's response to cocaine cues in males with a history of childhood trauma (and this reduction was associated with decreased craving). However, it increased amygdala cue reactivity in females with a history of childhood trauma. Males and females without trauma showed no reduction in amygdala activity after oxytocin treatment. Thus, oxytocin may be an effective therapy for cocaine-dependent men, while further research is needed to explore its use in women (Joseph et al., 2019). Another study evaluated the effectiveness of intranasal oxytocin (40 IU) in reducing the neuroendocrine and emotional responses induced by the Trier Social Stress Test in both males and females with CUD. After stress exposure, women reported greater levels of stress than men. Treatment with oxytocin did not significantly affect craving or stress, but it did blunt the cortisol response in women with CUD. This study also revealed that higher levels of endogenous progesterone were associated with a reduced craving response in women (Sherman et al., 2020). Together, these results suggest that social stress poses a higher risk of relapse in women with CUD and that treatment with progesterone or oxytocin may mitigate the pro-relapse effects of stress.

### 7.2 Preclinical studies

Several preclinical studies that included female subjects have evaluated the efficacy of pharmacotherapies in preventing the effects of stress on cocaine sensitivity. For instance, administration of rimonabant, a CB1R inverse agonist/antagonist, reduced the increase in cocaine SA induced by footshock in both male and female rats. However, the high dose of rimonabant also decreased cocaine SA in non-stressed females. These results suggest that CB1R activity contributes to the effects of stress on cocaine SA, likely via endocannabinoid signaling in the VTA (Gaulden et al., 2025). Similarly, antagonism of orexin-1 receptors blocked yohimbine-induced reinstatement of cocaine SA in both sexes (Zhou et al., 2012), while allopregnanolone blocked yohimbine-induced reinstatement in females but not in males (Anker and Carroll, 2010). Inhibiting RKBP5, a co-chaperone of the glucocorticoid receptor that regulates the negative feedback of the HPA axis, reduced stress-induced reinstatement in male rats and in female rats during the metestrus or diestrus phases (but not in proestrus or estrus) during the reinstatement test (Connelly et al., 2020); in addition, a CRF1 receptor antagonist attenuated the increase in cocaine SA observed on the first day of extinction, particularly in female rats (Cason et al., 2016). Inhibition of the HPA axis negative feedback also diminished anxiety-like symptoms induced by withdrawal from cocaine SA (Connelly et al., 2020). Finally, oxytocin appears to play a similar role in cocaine-seeking behavior in both male and female rats. In males, oxytocin (0.3–3 mg/kg) attenuated cocaine SA as well as priming- or cue-induced reinstatement of cocaine seeking after extinction (Zhou et al., 2014). In females, oxytocin attenuated cocaine SA and cue-induced reinstatement at the same doses that were effective in males, but only reduced cocaine-induced hyperactivity in females (0.1–3 mg/kg) and not in males (Leong et al., 2016). Administration of oxytocin also decreased cocaine SA on the first day of extinction, delayed extinction of SA, and reduced the cue-induced reinstatement of cocaine seeking in both sexes (Kohtz et al., 2018). Consistent with these behavioral results, neurons in the paraventricular and supraoptic nuclei in both sexes exhibited less activity on the first day of withdrawal (home cage) and during the first extinction session than control rats, although cocaine exposure increased oxytocinergic-expressing neurons in the supraoptic nucleus (Kohtz et al., 2018). It is important to note that the effectiveness of guanfacine has not been tested in female rodents. However, this drug has been shown to decrease stress-induced reinstatement of cocaine-seeking behavior in male rodents using the CPP paradigm (Mantsch et al., 2010; Vranjkovic et al., 2012). Furthermore, clinical studies have suggested that guanfacine may be more effective in treating several aspects of CUD in females.

## 8 Drug addiction stages

The complex etiology of cocaine-use disorder (CUD) involves the interaction of biological variables, environmental factors, and cocaine use (e.g., genes, personality, social context, and neuroadaptations). Stress is one of the main risk factors for cocaine users; therefore, it is critical to understand how stressful life events contribute to the initiation, maintenance, and development of cocaine use and CUD. Stress is also a key factor that increases the propensity for drug relapse, which is a persistent obstacle to the treatment of CUD. Stress triggers drug craving in abstinent cocaine users and interacts with other relapse triggers, such as exposure to small doses of cocaine or cocaine-associated cues. Sex, on the other hand, is a critical biological variable in the transition from recreational cocaine use to addiction. The interaction between sex, stress, and cocaine use may explain why females appear to be more vulnerable to CUD.

Individuals with drug addiction disorders, including CUD, experience a spiral of addiction consisting of three distinct stages (Compton et al., 2022). During the Binge/Intoxication stage, which primarily involves the brain reward system (mesolimbic DA from VTA to NAcc and basal ganglia), individuals consume the drug because it provides them with pleasure due to its rewarding effects. During the Withdrawal/Negative Affect stage, which involves stress hormones and the extended amygdala, individuals experience a negative emotional state when they are not using the drug. The last stage is Preoccupation/Anticipation, which involves interactions between the PFC, the extended amygdala, and the brain reward system. During this stage, individuals seek the drug again after a period of abstinence. Chronic cocaine exposure induces changes in limbic and prefrontal brain areas. Changes in the mesolimbic system affect DA plasticity, which facilitates the learning of strong associations between drug reward and the cues that acquire incentive salience. Conversely, deficits in DA and activation of stress systems induce dysphoria during withdrawal and craving. Drug-associated cues trigger the anticipation of reward and heighten craving during abstinence. Control of drug consumption shifts to structures associated with automatic, habitual behaviors, such as the nigrostriatal system and the basal ganglia. Ultimately, chronic drug exposure weakens the ability of the PFC to inhibit compulsive drug consumption.

Self-reports from cocaine users suggest that the transition to cocaine dependence occurs more quickly among women, who also report more intense cravings in response to cocaine-associated cues than men. However, there is little experimental evidence supporting sex differences in cocaine craving and relapse (McLaughlin and Fuchs, 2020). In addition, population-based surveys indicate that sex differences in the timeline for developing CUD are often influenced by sociocultural factors and variations in the likelihood of developing CUD and seeking treatment for the disorder. Studies with non-treatment-seeking populations report mixed results regarding the telescoping effect, which is more evident in studies with treatment-seeking individuals who have presumably developed more severe CUD (Towers et al., 2023a). In this context, a population-level study showed that adolescent and young adult females were less likely than males to have a mild-to-moderate illicit drug-use disorder, but equally or even more likely to develop a severe illicit drug-use disorder (Cotto et al., 2010). Furthermore, preclinical studies strongly suggest that females are more vulnerable to CUD. For example, female rodents develop addiction-like features more quickly than male rodents, particularly in the SA paradigm. This includes faster acquisition of the operant response to obtain cocaine, higher cocaine intake and escalation of cocaine SA, greater motivation for cocaine, continued drug-seeking behavior in the absence of reward, and, when responding has negative consequences, enhanced vulnerability to incubation and a greater risk of reinstatement of cocaine-seeking behavior when exposed to low doses of cocaine, cocaine-paired cues, or stress.

As discussed, both human and preclinical studies have shown that chronic cocaine exposure leads to sex-specific changes in endocrine and neural responses to cocaine-associated cues and stress, as well as in executive functions, including inhibitory control. These changes put women at a high risk of relapse and impede successful treatment. Women with CUD experience complex interactions when drug cues and stress triggers are present. Those with high scores on a self-report questionnaire measuring antecedents of relapse (negative, positive, and temptation situational drug use) exhibit greater cortisol responses to cue reactivity tasks after receiving yohimbine (Campbell et al., 2019). A recent study reported no association between self-perceived stress coping ability and treatment outcomes for female participants, while these variables were positively associated in males, suggesting that women face additional barriers to treatment engagement (Schick et al., 2024). Thus, understanding the specific cocaine- and stress-induced brain alterations that occur in females could help develop effective, sex-tailored therapeutic approaches. One potentially interesting method is to administer repeated transcranial magnetic stimulation to the brain areas affected in women with CUD. Preclinical studies have demonstrated that exposure to stressful life events at different developmental stages increases the effects of cocaine on the SA paradigm more significantly in females than in males; however, results regarding the effects of cocaine on other behaviors (CPP, hyperactivity, and behavioral sensitization) are inconclusive. These findings highlight the importance of studying the role of stress in male and female animals across different developmental stages using a variety of behavioral paradigms to evaluate the effects of cocaine.

**Figure 2:**
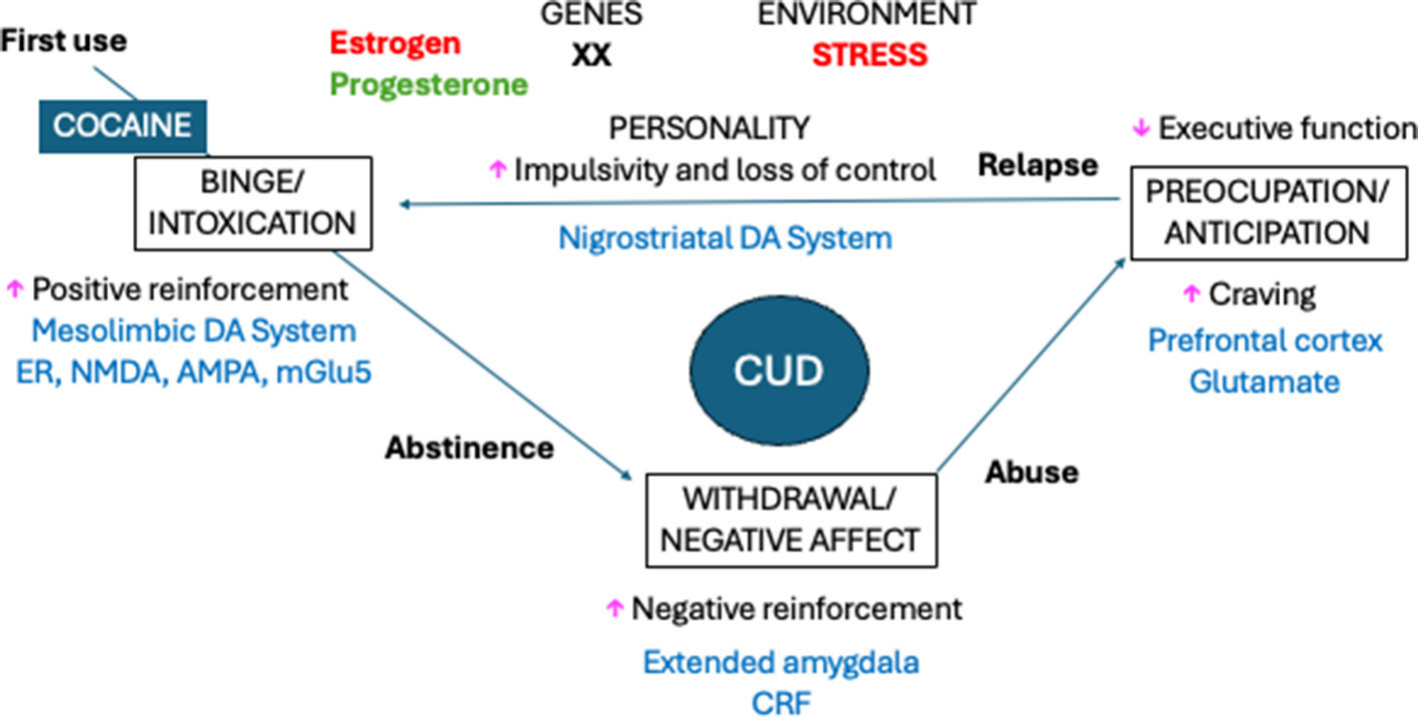
Potential behavioral and neurobiological mechanisms underlying the vulnerability of females to develop a cocaine-use disorder (CUD). DA, dopamine; ER, estradiol receptor; NMDA and AMPA, ionotropic glutamate receptors; mGlu5, metabotropic glutamate receptor type 5; CRF, corticotropin-releasing factor. Red indicates risk factors; green indicates protective factors; pink indicates a higher effect in comparison to males; blue indicates brain substrates. Compared to males, female rodents showed faster acquisition of cocaine self-administration, higher cocaine intake, faster escalation, higher binge use, greater motivation for cocaine, faster incubation, higher stress-induced reinstatement of cocaine seeking, and greater compulsivity. All these factors contribute to a faster development of the addiction-like phenotype in females.

Figure 2 shows that multiple elements of the addiction cycle can be modulated by ovarian hormones. Stress also plays an important role in sex-related differences in the development of cocaine addiction. The progression from initial cocaine use to repeated binge consumption involves the drug's positive reinforcing effects, which are mediated by the mesolimbic dopaminergic system. This system is influenced by the glutamatergic system (NMDA, AMPA, and metabotropic receptors) and estrogens. Sex differences in these neurotransmitter systems and estradiol receptor (ER) activation could increase the hedonic effects of cocaine in females. Preclinical studies support this hypothesis, indicating that female rodents acquire SA faster, have higher cocaine intake, and exhibit escalation and binge use more quickly. The negative affect induced by withdrawal involves negative reinforcement mainly mediated by the activation of CRF receptors in the extended amygdala. Clinical and preclinical evidence suggests that females experience greater negative affect during cocaine abstinence, which could facilitate abuse. Preclinical studies have demonstrated that females develop an addiction-like phenotype faster, with higher motivation for cocaine, enhanced craving, and faster incubation. Alterations in the glutamatergic system in the prefrontal cortex mediate craving and impairments in executive functions that could also be more pronounced in females, facilitating relapse to drug use after a period of abstinence. Preclinical studies have shown that females are more vulnerable to reinstatement, particularly stress-induced reinstatement of self-administration (SA). Individual factors, such as sex and personality, also modulate vulnerability to developing a CUD. Impulsivity, loss of control, and compulsivity, which are mediated by activation of the nigrostriatal dopaminergic system, may be more pronounced in females. Similarly, estrogen acts as a risk factor, while progesterone may protect against CUD development. Finally, exposure to stress at different developmental stages acts as an environmental factor that increases the risk of developing a CUD. Clinical and preclinical studies suggest that stressful events may have a greater influence on female vulnerability to cocaine.

## 9 Implications of the results obtained in preclinical studies for the prevention and treatment of CUD in women

Preclinical studies have provided evidence that female rodents are more vulnerable to the development of cocaine craving and relapse. These studies have also enhanced our understanding of the mechanisms by which stress increases the reinstatement of cocaine-seeking behavior and have tested the effectiveness of several pharmacological compounds in preventing relapse (McLaughlin and Fuchs, 2020). The influence of neuroendocrine mechanisms, ovarian hormones, and the estrous cycle has been reported in stress-induced reinstatement (Anker and Carroll, 2010) as well as in stress-potentiated priming (Doncheck et al., 2020) and cue-induced reinstatement (Feltenstein et al., 2011) of cocaine-seeking behavior. Estradiol may be a risk factor for cocaine-use disorder in women, including vulnerability to stress, while progesterone and allopregnanolone could be potential treatment options (Allen et al., 2016; Milivojevic et al., 2016). In this context, future studies should examine in more detail the influence of the menstrual cycle on relapse to cocaine seeking in women with CUD. Combining pharmacological strategies that control hormonal fluctuations with other anti-relapse medications could be effective. Signaling at CRF receptors may be involved in the increased drug-seeking behavior during initial abstinence, when stress levels are high; preclinical studies suggest that antagonizing these receptors (Cason et al., 2016), manipulating the HPA axis (Connelly et al., 2020), or administering the alpha-2 adrenergic agonist guanfacine (Fox et al., 2014; Milivojevic et al., 2017) could be useful pharmacological approaches for treating CUD in women. Additionally, oxytocin may reduce cocaine-seeking during initial abstinence and in response to cocaine-associated cues (Che et al., 2021; Kohtz et al., 2018), as might the pharmacological modulation of the endocannabinoid system (Doncheck et al., 2020; Gaulden et al., 2025).

In addition to pharmacological treatments, several environmental strategies can be used to prevent or reduce the negative effects of stress on CUD. Preclinical studies have shown that social housing and physical exercise can reverse the effects of isolation stress on the escalation of cocaine SA and CPP reinstatement, respectively. Specifically, paired housing was shown to attenuate the increased motivation to take cocaine induced by isolation during late adolescence in females, but not in males (Westenbroek et al., 2013). In addition, the reinstatement of cocaine CPP induced by immobilization stress in young adult female rats was prevented by chronic exposure to aerobic exercise (Robison et al., 2018b). Similar protective effects of exercise were observed in social defeat-induced potentiation of cocaine CPP in male mice (Calpe-López et al., 2022). The results of these studies suggest that environmental interventions induce adaptations in stress pathways that counteract the negative effects of stressful life events on cocaine addiction in females.

## 10 Discussion: similarities and differences between clinical and preclinical studies

A comprehensive comparison between the results of clinical and preclinical studies focused on sex differences in cocaine vulnerability modulated by stress shows more similarities than differences, even though the two lines of research have distinct methodologies and scopes. While clinical trials use indirect techniques such as neuroimaging or biomarkers, studies using animal models enable direct analysis of hormones, neurotransmitters, or receptors.

In general, there is consistent evidence suggesting that females are more susceptible to the reinforcing and relapse-inducing effects of cocaine when exposed to stress. Thus, the clinical studies report that women progress more rapidly to CUD, exhibit higher craving and relapse rates, and experience greater subjective stress responses compared to men, especially during early abstinence (Becker et al., 2017; Martin et al., 2021; Peart et al., 2022; Baker et al., 2022). Similarly, female rats and mice show heightened cocaine intake, faster acquisition of self-administration, greater motivation, and more robust relapse-like behaviors, particularly following long-access self-administration protocols and stress exposure (Lynch and Carroll, 1999; Towers et al., 2021; Thomas and Becker, 2019). Additionally, females are more sensitive to reinstatement triggered by stress-related stimuli like yohimbine or footshock (Anker and Carroll, 2010; Buffalari et al., 2012).

However, findings across clinical studies are not always consistent. For example, most studies reported no significant sex differences in craving and did not confirm greater vulnerability to relapse in women (Nicolas et al., 2022). In contrast, preclinical studies in rodents provide more consistent evidence, likely due to the easier control of influencing variables in these studies.

These sex-specific vulnerabilities are strongly influenced by hormonal factors. In both humans and rodents, estradiol enhances the rewarding and motivational effects of cocaine, while progesterone tends to reduce them (Evans and Foltin, 2010; Satta et al., 2018; Torres, 2022). Moreover, female rodents in the estrus phase show greater dopaminergic responses to cocaine in the nucleus accumbens (Calipari et al., 2017; Hersey et al., 2023).

There are also similarities in the results of preclinical and clinical studies regarding the response to stress across sexes. Women with CUD show blunted cortisol responses but elevated subjective stress and craving after psychosocial stress (Waldrop et al., 2010; Baker et al., 2022). Similarly, female rodents exhibit higher corticosterone levels and greater sensitivity to CRF and noradrenergic signaling (McRae-Clark et al., 2017; Martin et al., 2021). Notably, exposure to stress during critical developmental periods (e.g., prenatal, early life, and adolescence) increases vulnerability to cocaine use in females more than in males, particularly after long-term or extended exposure (Thomas and Becker, 2019; Arenas et al., 2022; Di Segni et al., 2019).

Nevertheless, the clinical findings are again less consistent, likely due to ethical limitations, comorbidities, and social influences, which are absent in controlled animal models. Conversely, preclinical studies offer mechanistic insights but may lack ecological validity for translation to human populations.

Both approaches agree that women and female animals are more vulnerable to the influence of stress on cocaine addiction, although this vulnerability is better characterized and evidenced in animal models. This is largely due to increased experimental control and the possibility of intervening at specific points in development in preclinical studies. Clinical studies, on the other hand, reflect a more complex reality influenced by social, psychological, and cultural factors.

In conclusion, while both clinical and preclinical research recognize sex differences, the evidence is more robust in animal models, underlining the need for greater inclusion of sex as a biological variable in both human and animal studies to improve treatment and prevention strategies for CUD (Martin et al., 2021; Becker et al., 2017).

## 11 Limitations of the review

We did not follow the PRISMA method when conducting this review, and our search was limited to the PubMed database because our goal is to provide a thorough analysis rather than a systematic review of clinical and preclinical studies comparing the effects of cocaine and how stress modulates these effects in both sexes. Additionally, we focus more on sex differences in the influence of stress exposure at different ages than on sex differences in other aspects of CUD or alterations induced by cocaine exposure in preclinical models.

Differences between species in the neurobiology of cocaine response, particularly in brain structures involved (cortical vs. subcortical areas) and the function of dopaminergic systems, as well as variations in cyclic fluctuations of sexual hormones, may influence the interpretation of findings observed in the studies reported in this review. Phylogenetic proximity to humans is greater in monkeys than in rats and mice, which could help explain the physiological differences between species. A limitation of the present study is the exclusion of research on non-human primates that could provide insights into the influence of stress on vulnerability to cocaine addiction with high translational value. Monkeys and humans share greater similarities than rodents in terms of cortico-striatal circuitry, behavioral complexity, higher-level cognitive functions, and social structures. However, research on the role of stress in cocaine addiction in non-human primates is limited. In fact, we found only two studies when searching for “stress,” “cocaine,” and “sex differences.” One of these studies evaluated the consequences of early-life stress (maltreatment) on sensitivity to the reinforcing effects of cocaine in late-adolescent male and female rhesus monkeys. The control male monkeys required more days to acquire cocaine SA than the maltreated males and both groups of females. These results suggest that early stress exposure enhances the vulnerability of late-adolescent male monkeys to cocaine and that non-stressed females are more sensitive to the reinforcing effects of this drug than males (Wakeford et al., 2019). Previous studies have also shown that females have higher PR breakpoints for cocaine SA than males (Mello et al., 2007). The greater vulnerability of female monkeys to cocaine compared to males aligns with findings observed in female rodents and some studies involving women. Another study compared the acquisition of cocaine SA in male and female monkeys living in stable social groups with different social ranks (subordinate vs. dominant). Social subordination is a chronic stress state, so subordinate monkeys of both sexes acquired cocaine SA at lower doses than their dominant counterparts (Johnson et al., 2024). The finding that chronic stress increases vulnerability to cocaine has also been observed in humans and rodents, but the lack of sex differences in stress effects is more consistent with clinical study results.

Although rats and mice are widely used to study the influence of stress on addiction, and there are well-validated models for many aspects of addictive behavior (e.g., drug reward, abstinence, craving, and relapse), these models do not directly translate to humans. Instead, the results obtained from rodent models must be confirmed in non-human primates or humans.

## 12 Future directions and conclusion

The present work sheds light on several gaps in the current knowledge regarding the influence of sex on the effects of stress exposure and vulnerability to cocaine.

We have identified several clinical studies conducted exclusively with crack cocaine-dependent women to explore the influence of early-life stress and the neurobiological mechanisms involved. These studies compared crack cocaine-dependent women with and without a history of physical neglect, maltreatment, or abuse in childhood (and sometimes also included healthy women). It would be interesting to study the role of early-life stress in crack vulnerability in men and in rodents of both sexes.

Future preclinical research should prioritize the study of the effects of different stress protocols, the use of early and late adolescent subjects, hormonal status tracking, the neurobiological substrates involved, and the potential pharmacological and environmental strategies that can prevent or reduce the effects of stress, particularly in females.

In conclusion, sex has long been overlooked in clinical and preclinical research on drug addiction, including CUD. However, there is growing evidence that both female rodents and women are more vulnerable than their male counterparts to the behavioral and neurochemical changes that characterize the different phases of the addictive cycle. Estradiol acts as a risk factor for the development of CUD. Furthermore, stress during different developmental stages appears to amplify the effects of cocaine, particularly in females, thus contributing to their heightened susceptibility to cocaine addiction. The influence of sex should be considered when conducting research in this field and when selecting strategies for the prevention and treatment of CUD, including those targeting stress reduction.
